# Caffeic Acid and Human Health: Evidence-Based Roles in Disease Prevention and Treatment

**DOI:** 10.3390/ijms27114719

**Published:** 2026-05-23

**Authors:** Saleh A. Almatroodi, Arshad Husain Rahmani

**Affiliations:** Department of Medical Laboratories, College of Applied Medical Sciences, Qassim University, Buraydah 51452, Saudi Arabia; smtrody@qu.edu.sa

**Keywords:** caffeic acid, antioxidant activity, anti-inflammatory activity, pathogenesis, chronic disease, cancer

## Abstract

Caffeic acid (CA) is a phenolic compound commonly found in fruits, vegetables, and coffee, with preclinical evidence demonstrating its important role in disease management through different mechanisms of action. This review aimed to explore CA’s pharmacological effects in different pathological conditions, and sources were retrieved by using databases like PubMed, Scopus, Google Scholar, and Web of Science and based on preclinical studies. CA notably protects cells and tissues from oxidative stress and inflammation, highlighting its therapeutic role in the management of pathogenesis. The neuroprotective, cardioprotective, hepatoprotective, anti-microbial, and anti-obesity effects are reported through in vitro and in vivo studies. Moreover, its anticancer effects are linked to modulation of cell signaling pathways, together with angiogenesis, cell cycle, apoptosis, and the PI3K/Akt pathway. This article explores how caffeic acid influences health conditions, providing a comprehensive overview of its effects on disease processes. Reviewing the literature aims to enhance the understanding of caffeic acid’s role in disease management and as a natural therapeutic agent. Although several studies demonstrate the anticancer effects and its role in the management of various pathological conditions, most of the existing evidence is based on in vitro, in vivo, and xenograft models. Moreover, many natural compounds, including CA, that exhibit activity in preclinical settings fail to translate into clinical applications, due to restrictions of poor bioavailability, toxicity, rapid metabolism, and differences in the tumor microenvironment. Thus, future studies should emphasize well-designed in vivo studies as well as controlled clinical trials to better describe CA’s safety, efficacy, mechanism of action, and therapeutic application in humans. Further investigation of its interactions with other therapeutic agents may offer insights into synergistic effects that enhance treatment efficacy. Overall, a more comprehensive understanding of this compound will be indispensable for its development as a therapeutic agent in the treatment of chronic disease.

## 1. Introduction

Medicinal and aromatic plants, particularly those utilized in ethnopharmacology, have served as a natural source of remedies as well as healthcare [1,2,3]. About 64% of the worldwide population depend on traditional medicine for their health needs [4]. Natural products provide a diverse array of components, together with flavonoids. Flavonoids are substantial secondary metabolites found in many sources such as fruits, flowers, vegetables, nuts, stems, cereals, herbs, and seeds [5]. To date, over 10,000 flavonoid compounds have been identified and isolated [6]. Moreover, natural products and bioactive compounds, including flavonoids, are vital in various disease processes, working through mechanisms like antioxidant and anti-inflammatory effects [7,8,9,10].

Caffeic acid is produced from the secondary metabolism of various vegetables [11,12,13,14], and it stands out as the principal hydroxycinnamic acid present in the human diet [11,13,14,15]. Its role in various aspects of pathological conditions, mainly based on preclinical studies, has been documented through modulation of various biological activities. A study examined the protective role of CA against neuronal apoptosis, oxidative damage, and cognitive impairment in a D-galactose–induced rat model of brain aging. Rats treated with D-gal alone showed substantial memory impairment, accompanied by scavenging enzyme activities and decreased Bcl-2expression. Moreover, D-gal administration evidently increased the number of p21-positive cells in the subgranular zone of the hippocampal dentate gyrus, along with elevated Bax and caspase-3 protein levels and higher malondialdehyde (MDA) concentrations. By contrast, CA administration reduced these effects [16]. Another study investigated the impact of CA on lipid profile alterations and endothelial function in a rat model induced by an atherogenic diet (Ath-). Results indicated that CA treatment led to an improved lipid profile and reduced oxidative stress. Aortic staining demonstrated a noteworthy decrease in the presence of atherosclerotic lesions [17].

A study focused on assessing the effects of CA against testicular injury induced by arsenic (As) in mice. The outcomes showed a noteworthy decrease in testicular FRAP, glutathione peroxidase, and superoxide dismutase levels, along with lower plasma concentrations of dihydrotestosterone and testosterone in mice exposed to arsenic when compared to the control group. Additionally, arsenic exposure caused histopathological and morphological changes in the testis, which included hyperemia, thickening of the epithelial cell layers in the seminiferous tubules, a reduction in tubular diameter, and necrosis of Leydig cells. The simultaneous administration of caffeic acid (CA) alongside arsenic (As) resulted in an increase in glutathione peroxidase, testosterone, and ferric reducing antioxidant power and dihydrotestosterone levels. Additionally, it reduced malondialdehyde levels (MDA) and mitigated histopathological changes to a degree where they were not significantly different from those noticed in the control group [18]. This review summarizes a comprehensive overview of caffeic acid, including its plant sources, pharmacokinetics, biological activities, and nano-formulation approaches. A thorough understanding of caffeic acid will assist researchers in directing future studies, promoting its development and application in disease management, which requires further preclinical and clinical validation.

## 2. Methodology

A comprehensive literature search was conducted as part of this narrative review through electronic databases, using PubMed, Scopus, Google Scholar, and Web of Science, to evaluate the therapeutic role of caffeic acid across various pathological conditions. The search covered studies published from November 1997 to February 2026. The search strategy incorporated combinations of keywords such as caffeic acid, pharmacological activity, antioxidant, anti-inflammatory, antidiabetic, hepatoprotective, cardioprotective, neuroprotective, cell signaling pathways, digestive system, respiratory system, anti-arthritis, anti-obesity, wound healing, and antimicrobial activity. In addition, terms related to caffeic acid, cancer, and cell signaling pathways were searched. Additional keywords such as synergistic effects and nano-formulation were also used to compile emerging therapeutic applications. The inclusion criteria involved research articles (in vitro and in vivo studies), clinical trials, and review articles focusing on caffeic acid and its therapeutic effects. Exclusion criteria comprised conference abstracts, editorials, duplicate publications, and non-English articles. A total of 199 articles were initially searched in the electronic database. After the removal of duplicate studies, 182 articles were considered for data compilation. Titles and abstracts of published articles were first checked for relevance, and then full-text evaluation was conducted to determine eligibility for inclusion in this narrative review.

This manuscript used AI-based tools, including ChatGPT (GPT-5.5) and Grammarly (pro version), to rephrase and improve the language.

## 3. Structure, Sources, Pharmacokinetics, and Bioavailability

**Figure 1:**
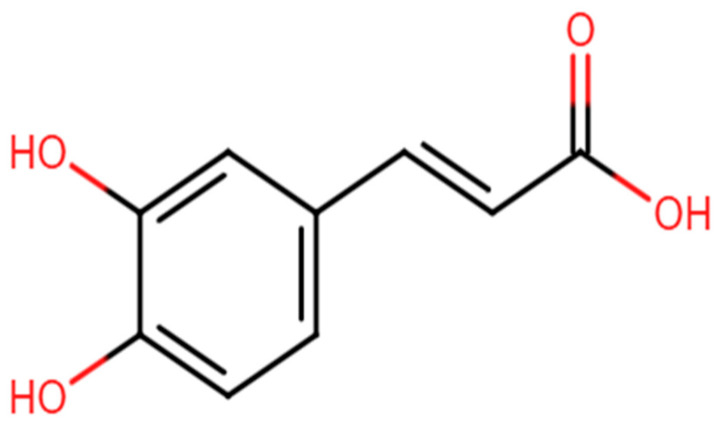
Caffeic acid chemical structure. The chemical structure was generated using the online website (https://www.rcsb.org/search/chemical), through the “Draw or Edit Chemical Structure Marvin JS by Chemaxon” (accessed on 25 March 2026).

Caffeic acid is an organic compound (Figure 1) with the chemical formula C9H8O4 and a molecular weight of 180.15. It features an aromatic core with a three-carbon unsaturated chain substituted at position 1, which includes a carboxylic group. Additionally, it possesses two hydroxyl groups at positions 4 and 5. Caffeic acid is classified as a hydroxycinnamic acid, which is characterized by its aromatic acids with a C6–C3 skeleton [19].

**Figure 2:**
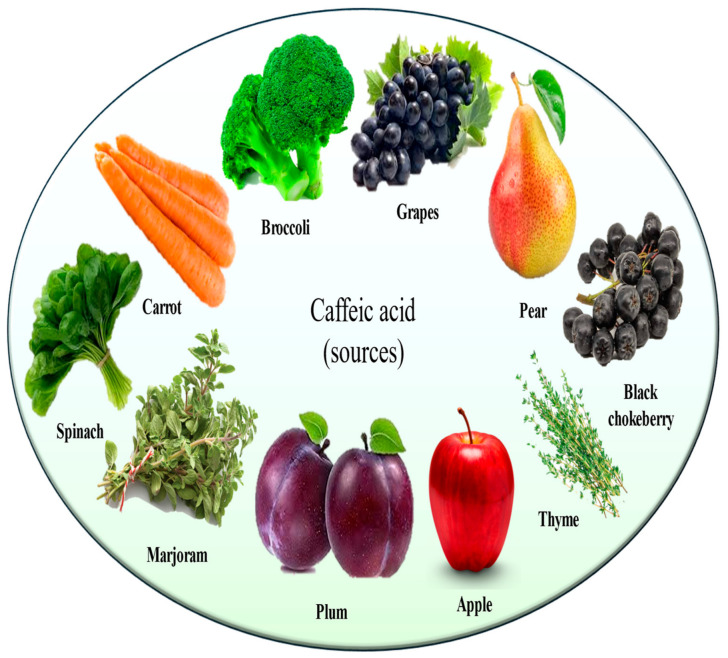
Natural dietary sources of caffeic acid. Caffeic acid is present in foods, including fruits (pear, grapes, plum, apple, black chokeberry), vegetables (carrot, spinach and broccoli), and herbs (marjoram and thyme).

The chemical synthesis of caffeic acid generally uses o-hydroxybenzaldehyde (3,4dihydroxybenzaldehyde) as well as acrylic acid as the chief starting materials. Then esterification, aldol condensation, and decarboxylation are carried out to get CA [20]. Caffeic acid occurs broadly in fruits and vegetables (Figure 2). Caffeic acid occurs extensively in plant-derived foods, with especially high levels in seeds particularly in found in coffee. It is also present in a variety of vegetables, fruits, olive oil, grains, and traditional Chinese medicinal materials [21,22,23,24,25,26,27]. Potato, carrots, artichokes, pot-grown basil, black tea, coffee, apple juice, orange juice, grapefruits, and buckwheat grits are source of caffeic acid [28,29,30]. Sources and content of caffeic in different food items as milligrams (mg) per 100 g is presented in Table 1.

Caffeic acid is absorbed in the body via multiple mechanisms. One route involves passive diffusion in the stomach, where CA exists predominantly in a non-ionized form due to the acidic environment [31,32,33]. In addition, a limited amount of caffeic acid is taken up by enterocytes lining the gastrointestinal tract [33,34]. Another absorption mechanism occurs in the small intestine via active transport mediated by sodium-dependent channels [32]. Furthermore, microbial esterases degrade the ester group of caffeic acid, a free form of caffeic acid to be absorbed across the intestinal mucosa [31]. The absorption as well as metabolism in the small intestine of caffeic acid were studied in rats. The net absorption reported was 19.5% of the perfused caffeic. A minor portion of the perfused caffeic acid was metabolized in the intestinal wall as well as secreted back into the gut lumen in the form of ferulic acid [34]. Another study examined the absolute bioavailability of CA in rats, as well as its intestinal absorption characteristics. A two-compartment pharmacokinetic model was found to be the most effective for demonstrating the pharmacokinetics of CA following both IV and IG administration. The Fabs of CA was noted to be 14.7%, whereas its intestinal absorption rate was 12.4%. The values of Papp A to B and Papp B to A for CA remained stable in spite of changes in its concentration. The efflux ratio detected in this study was larger than 2.0, showing that CA undergoes active transport. Moreover, CA demonstrated poor permeability across Caco-2 cells, which contributes to its restricted intestinal absorption as well as low oral bioavailability in rats [35].

The pharmacokinetics of caffeic acid (CA) were investigated in rabbits using three different doses: 5, 10, and 25 mg kg^−1^. The outcomes revealed that total body clearance and the elimination rate constant from the central compartment (k10) were greater after the 5 mg kg^−1^ dose compared to the other two doses. Additionally, the terminal elimination half-life (beta half-life) and mean residence time (MRT) were shorter following the 5 mg kg^−1^ dose compared to the other doses. The AUC values showed a linear increase with doses within the 10–25 mg kg^−1^ range. Most of the unchanged caffeic acid was cleared through urine within 2 h. The percentages of of unchanged caffeic acid excreted in urine after administering doses of 5, 10, and 25 mg kg^−1^ were 63.4%, 60.0%, and 55.4%, respectively [36]. Caffeic acid exhibits low oral bioavailability in rats, with an absolute bioavailability (Fabs) of 14.7% and an intestinal absorption of 12.4%. This poor systemic availability is attributed to its limited intestinal uptake and weak permeability across Caco-2 cells [37].

Although caffeic acid is present in a variety of dietary sources such as coffee, fruits, and vegetables, its systemic exposure after normal dietary intake is relatively limited due to rapid absorption and fast elimination. The rapid and wide-ranging metabolism results in low plasma concentrations and fast elimination from circulation. The studies based on absorption, metabolism, and excretion of caffeic acid are limited. Free caffeic acid was rapidly detected in plasma following the consumption of red wine [38,39]. Astudy evaluated the bioavailability of CA and changes in plasma antioxidant status after red wine consumption. Five healthy male participants consumed 100, 200, and 300 mL of red wine, providing approximately 0.9, 1.8, and 2.7 mg of caffeic acid, correspondingly. Plasma samples were collected at different times in a 0–300 min period and analyzed for caffeic acid content and total antioxidant capacity. Both plasma caffeic acid levels and antioxidant activity were dose-dependent, and the *C*_max_ was reached at about 60 min after red wine intake. The findings advocate that caffeic acid is bioavailable in humans and may contribute to the antioxidant capacity of plasma [40]. The CA-based pharmacokinetics of methanol extract of seed of *Syzygium cumini* L. in rats was investigated. It was reported that following the extract administration, CA attained maximum plasma concentration (5.96 ± 0.49 μg/mL) in 1.0 h, which was the time to attain maximum concentration (T_max_). The outcomes indicate that CA absorption from the oral route is quick, but lower amounts are absorbed [41].

## 4. Experimental Evidence of the Impact of Caffeic Acid in Human Health and Disease Processes

Caffeic acid may play a vital role in disease-related processes by multiple mechanisms. Preclinical evidence from in vitro as well as in vivo studies demonstrates its possible beneficial effects on the progression of numerous chronic diseases. This review aims to explore the various effects of caffeic acid across multiple health conditions and provide an outline of its involvement in disease-related processes. By reviewing the existing literature, this work aims to improve the understanding of how caffeic acid contributes to managing pathological conditions, which needs further clinical validation. Moreover, this review describes the roles of caffeic acid in different pathological conditions based on previous preclinical evidence, emphasizing its significance in health and disease management.

### 4.1. Antioxidant Activity

Caffeic acid exhibits strong antioxidant activity through multiple complementary mechanisms. The antioxidant activity of caffeic acid is mainly associated with its ability to scavenge free radicals, suppress lipid peroxidation, and enhance endogenous antioxidant enzymes, activating the Nrf2/HO-1 signaling pathway. Through these mechanisms, caffeic acid contributes to the prevention of oxidative stress-associated pathological conditions. The protective effects of caffeic acid (CA) against IR-induced neuronal cell damage were observed. The findings advocate that CA covalently modifies active cysteine residues on the Keap1 protein, leading to conformational changes that disrupt the Keap1–Nrf2 interaction. This disruption promotes the translocation of Nrf2 into the nucleus, where it stimulates the expression of downstream cellular protective factors, including heme oxygenase-1 (HO-1), NAD(P)H: quinone oxidoreductase 1 (NQO1), and thioredoxin reductase-1 and other cellular protective factors, to show a role in countering radiation-induced neurological damage [42].

**Figure 3:**
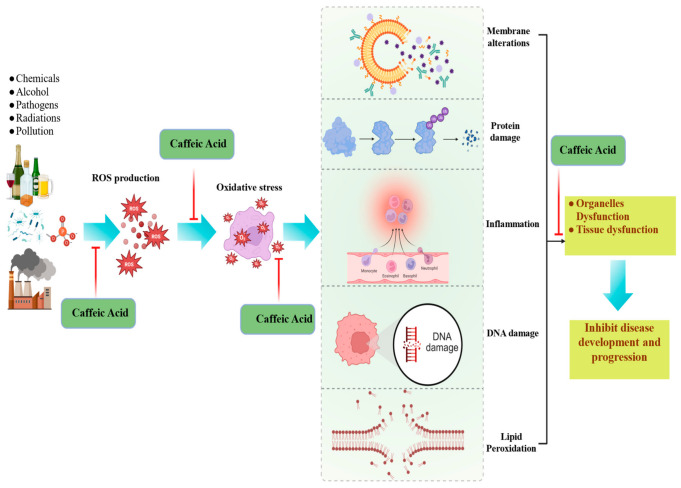
The antioxidant mechanisms of caffeic acid. Exposure by environmental and physiological stressors indoors increases excessive reactive oxygen species (ROS) generation and oxidative stress. Caffeic acid functions as an effective antioxidant through suppressing reactive oxygen species (ROS) generation and lessening oxidative stress. Through these activities, it protects cellular macromolecules and membrane integrity, reduces organelle dysfunction and tissue damage, and ultimately contributes to the prevention and progression of disease processes. The arrow shows downregulation/inhibition. The figure was created using BioRender, accessed on 29 December 2025. Rahmani AH. (2026). https://app.biorender.com.

The role of CA in the management of the disease process through antioxidant effects is demonstrated in Figure 3. Several studies clearly demonstrate the antioxidant effects of caffeic acid; some are discussed here.

The antioxidant activity of caffeic acid was estimated by using several in vitro assays, for example, ABTS radical scavenging, total antioxidant activity, and DPPH scavenging. The 10 and 30 µg/mL concentrations of CA suppressed lipid peroxidation in a linoleic acid emulsion by 68.2% as well as 75.8%, correspondingly. Similarly, it showed significant antioxidant effects through capably scavenging ABTS^+^ radicals as well as superoxide anion radicals [43]. The study aims to assess the in vitro antioxidant activity of CA in order to enhance its application and reliability in preventing damage caused by free radicals and other reactive species. The tests performed were as follows: Radical anion superoxide capture; capturing ability of hypochlorous acid crocin bleaching assay; H_2_O_2_ capture; SOD-like activity and capturing capacity of the ABTS•+/DPPH•. The antioxidant activity of CA was very close to the standards in all tests [44].

A study assessed the beneficial effects of both chlorogenic acid and caffeic acid using in vitro and in vivo models. In the in vitro analysis, caffeic acid exhibited stronger antioxidant activity compared to chlorogenic acid. Additionally, an intestinal ischemia–reperfusion model was used to assess their antioxidant effects in vivo. The results demonstrated that both caffeic acid and chlorogenic acid play protective roles against intestinal ischemia–reperfusion injury [45]. Another study reported that γ-irradiation of lymphocytes caused a dose-dependent rise in thiobarbituric acid–reactive substances and genetic damage, along with a noteworthy decline in antioxidant defenses. In contrast, pretreatment with caffeic acid effectively counteracted these radiation-induced alterations through its antioxidant effects [46].

### 4.2. Anti-Inflammatory Activity

Natural compounds have gathered substantial attention for their anti-inflammatory properties, which are attributed to several mechanisms that help to modulate inflammation. Caffeic acid has revealed noteworthy anti-inflammatory activity via several mechanisms such as inhibition of pro-inflammatory mediators and cell signaling molecules involved in inflammatory processes. Some findings based on the anti-inflammatory activity of caffeic acids are discussed here. A study reported that CA significantly reduced the production of TNF-α and IL-6 in fibroblast-like synoviocytes. Also, caffeic acid suggestively inhibited the expression of PGE_2_ and MMP-1 key mediators involved in the diseases of rheumatoid arthritis at both transcriptional and translational levels [47]. The study intended to investigate the ability of caffeic acid to influence mechanisms associated with intestinal inflammation. The outcomes showed the following: (i) caffeic acid targets cyclooxygenase-2 (COX-2), reduces its product PGE_2_, and decreases the biosynthesis of IL-8 in IL-1β-treated cells; (ii) it inhibits the formation of AGEs; and (iii) it exerts high chelating activity. In addition, caffeic acid exhibited only limited anti-ACE activity, and antioxidant and reducing capacities of caffeic acid were also detected [48]. A study examined the protective effects of caffeic acid (CA). In this experiment, mice with DSS-induced colitis were supplemented with 251 mg/kg of CA. The outcomes confirmed that CA treatment reduced the levels of pro-inflammatory cytokines as well as malondialdehyde (MDA), while increasing IL-10 and antioxidant enzyme levels in the serum. In addition, CA supplementation helped protect the intestinal barrier by increasing the expression of the Occludin gene [49]. The anti-inflammatory activity of caffeic acid was assessed using an HCl/EtOH-induced gastritis model and LPS-stimulated RAW264.7 macrophages. Caffeic acid markedly decreased nitric oxide (NO) and PGE_2_ production in LPS-treated macrophages and downregulated the mRNA expression of TNF-α, COX-2, and iNOS. In addition, CA alleviated HCl/EtOH-induced gastric injury by inhibiting key inflammatory signaling molecules, including IRAK1, JNK, and IRAK4. Thus, the data propose that CA works as an anti-inflammatory drug through suppressing IRAK1 as well as IRAK4 [50].

### 4.3. Anti-Diabetic Activity

Natural products and their bioactive constituents have demonstrated a noteworthy role in the prevention and management of diabetes mellitus [51,52,53], and caffeic acid has received substantial attention because of its promising anti-diabetic properties. Caffeic acid can influence several metabolic pathways involved in glucose homeostasis, improve insulin sensitivity, and regulate carbohydrate metabolism (Table 2).

A study’s findings reported that caffeic acid induced a substantial decrease in the glycosylated hemoglobin and blood glucose levels compared to the control group. The plasma insulin, leptin, and C-peptide levels in the caffeic acid group were higher, while the plasma glucagon levels were lower. Plasma insulin levels increased in response to caffeic acid, attributable to its antidegenerative activity in islets. Caffeic acid significantly enhanced the activity of glucokinase and its mRNA expression, as well as increased glycogen levels. At the same time, caffeic acid reduced both the activity and mRNA expression of glucose-6-phosphatase and phosphoenolpyruvate carboxykinase, along with lowering the expression of GLUT2 in the liver. Furthermore, caffeic acid enhanced the activities and gene expression of key antioxidant enzymes while decreasing the levels of TBARS and hydrogen peroxide in the erythrocytes and the liver of mice. These findings suggest that caffeic acid holds a considerable role as a natural antidiabetic agent [54].

The study found that CA decreased plasma glucose levels in a dose-dependent manner in streptozotocin (STZ)-diabetic rats, accompanied by an increase in plasma β-endorphin-like immunoreactivity (BER). These actions of CA were stopped by RS 17 056 or WB 4101 pretreatment at doses adequate to block alpha 1A-adrenoceptors. Moreover, naloxonazine and naloxone at doses effective for hindering opioid micro-receptors stopped the plasma glucose-lowering action of CA. It was noted that in a concentration-dependent way, CA improves β-endorphin-like immunoreactivity (BER) release from isolated rat adrenal medulla [55].

In another study, diabetes was experimentally induced in overnight-fasted rats through a single intraperitoneal injection of streptozotocin (STZ), followed by nicotinamide administration. After induction, the diabetic animals received oral treatment with caffeic acid as well as 18β-glycyrrhetinic acid. The diabetic rats showed elevated levels of liver and kidney function markers, total cholesterol, triglycerides, and malondialdehyde (MDA). Administration of either caffeic acid or 18β-glycyrrhetinic acid alone improved these biochemical alterations to some extent. However, the combined treatment of both compounds significantly restored the altered biochemical parameters in STZ-induced diabetic rats [56]. The antidiabetic effects of caffeic acid were examined in rats with type 2 diabetes induced by fructose and streptozotocin. The treatment with caffeic acid resulted in increased serum insulin levels, lowered blood glucose levels, and enhanced pancreatic β-cell function, glucose tolerance, and morphology [57]. A study was conducted to observe the anti-diabetic effects of caffeic acid in diabetic rats. It was observed that fasting blood glucose and MDA levels were increased, while insulin and antioxidant enzymes levels were reduced in diabetic rats. However, the diabetic rats treated with caffeic acid showed beneficial properties in all the biochemical parameters in diabetic rats [58]. The anti-diabetic effects of CA in a streptozotocin-induced diabetic rat model was investigated. CA was orally administered daily for five weeks. It was reported that CA caused noteworthy improvement in serum insulin level, and reduction in blood glucose level. CA caused antioxidant properties by increasing CAT, SOD, and GSH levels. Additionally, histological investigation of the pancreas showed normal islet morphology by administration of CA in diabetic rats [59]. A study finding reported that caffeic acid or 10-Dehydrogengardione inhibited MiR-122, hepatic FAS, as well as ACLY levels along with activation of p-AMPK. This afterward led to reduced plasma triglyceride, cholesterol levels, and improvement of blood glucose and describe their mechanisms as hypolipemic agents [60]. The effect of CA on serum lipid profiles as well as the atherogenic index in alloxan-induced diabetic rats was determined. Serum level of lipid parameters, fasting blood glucose (FBS), and the atherogenic index were determined. The study results showed that treatment with CA suggestively reduced the serum levels of fasting blood sugar, LDL-C, and total cholesterol in diabetic rats as compared with untreated diabetic animals. In addition, the atherogenic index was notably decreased following CA administration. The outcomes of this study designated that CA has valuable impact on serum lipid profile, blood glucose, and atherogenic index in type 1 diabetic rats [61].

**Table 2:** Antidiabetic effect of caffeic acid. The effects of CA on glucose regulation, lipid profiles, liver, kidney function tests, and insulin secretion. The table includes study type, dose, and outcomes.

Study Model	Dose	Outcomes	Refs.
STZ-induced diabetic rat model	3 mg/kg	° Plasma glucose levels decreased by CA	[55]
Streptozotocin-nicotinamide induced diabetes rat model	40 mg/kg	° The liver and kidney function tests decreased by CA ° Antioxidants status increased and lipid peroxidation decreased by CA	[56]
Fructose-streptozotocin-induced diabetic rat model	150, 300 mg/kg	° Blood glucose level reduced by CA	[57]
STZ-induced DM rat model	40 mg/kg	° Fasting blood glucose and MDA decreased by CA ° Insulin and antioxidant enzymes increased by CA	[58]
STZ-induced diabetic rat model	25, 35 mg/kg	° Blood glucose decreased and insulin level improved by CA	[59]
STZ-induced diabetes hyperlipidemic rat model	50 mg/kg	° CA groups decreased miR-122 expression ° CA increased p-AMPK activity ° CA reduced histopathological changes	[60]
Alloxan-induced diabetic rat model	50 mg/kg	° Serum blood glucose and lipid profile decreased by CA	[61]
Alloxan-induced diabetes rat model	50 mg/kg	° CA protects kidney and liver	[62]

### 4.4. Neuroprotective Effect

Caffeic acid exhibits neuroprotective effects through various mechanisms, as described in Table 3.

CA’s role in amyloid beta (Aβ_1-42_)-caused memory impairments and oxidative stress was evaluated. CA (50 mg/kg/day) was administered for two weeks to Alzheimer’s disease (AD) mice. Moreover, CA improved cognitive function and learning deficits and attenuated behavioral, biochemical, and histopathological changes. Caffeic acid significantly enhanced spatial learning, cognitive performance, and memory in an Alzheimer’s disease (AD) mouse model. The treatment also increased the expression of synaptic markers in the AD mouse model. Moreover, CA treatment also reduced BACE -1 and Aβ expression in the Aβ-induced AD mice model [63]. The study measured the impact of caffeic acid (CA) on two experimental depression models: chronic unpredictable mild stress (CUMS) as well as dexamethasone (DEXA). Administration of CA at 50 mg/kg produced anxiogenic effects in the CUMS model, which were related with elevated levels of hippocampus nitrite and glutamate. In contrast, the same dose exhibited anxiolytic activity in the DEXA model, accompanied by serotonin (5-HT) reduction. Furthermore, CA confirmed antidepressant-like activity in the CUMS model by enhancing hippocampal nitric oxide (NO) production, while in the DEXA model, its antidepressant-like effect was linked to a decrease in hippocampal glutamate levels [64].

The study inspected the protective role of caffeic acid on hippocampal neurogenesis and L-methionine (L-met)-induced cognitive deficits in rats. Findings advocate that diminished vascularization corresponded with reduced cell proliferation and lower neuronal survival. Furthermore, cell cycle arrest was elevated in the L-met-treated group. These outcomes demonstrated an association between spatial and recognition memory impairments. Notably, co-administration of caffeic acid effectively restored deterioration [65]. One study demonstrated that exposure to cadmium chloride (CdCl_2_) impaired cognitive performance and disrupted the activities of cholinesterase, purinergic enzymes, nitric oxide (NOx), monoamine oxidase, and arginase, along with antioxidant defenses. Treatment of cadmium-exposed rats with vitamin C and CAF improved cognitive function and normalized the altered enzymatic activities when compared to untreated cadmium-exposed animals. In the same vein, CAF administration plays a neuroprotective role in healthy rats, as demonstrated by improved cognitive function, increased antioxidant status, and decreased activity of enzymes associated with the progression of cognitive dysfunction, compared with healthy rats devoid of CAF [66]. The study based on result concluded that caffeic acid (20 and 40 mg) protect against L-methionine caused memory deficits [67]. Other results designate that caffeic acid effect on learning deficits in a model of AD by the oxidative stress and inflammation suppression through the p38 MAPK signaling pathway [68].

The study evaluates the effects of CA on learning deficits in a rat model of Alzheimer’s disease. First, CA was verified for in vitro anticholinesterase activity by homogenate of rat brain. Then, the in vivo antidementia role of CA was measured in aluminum chloride (AlCl3)-induced dementia in rats. It was found that CA administered to rats led to improved cognitive function, and the antidementia role of CA was established by the decrease in brain nitrite levels and AChE activity. Additionally, CA increased the reduced level of antioxidant enzymes in the brain. These results advocate for the antidementia effect of CA against AlCl3 -caused dementia [69].

### 4.5. Anti-Cancer Activity

**Figure 4:**
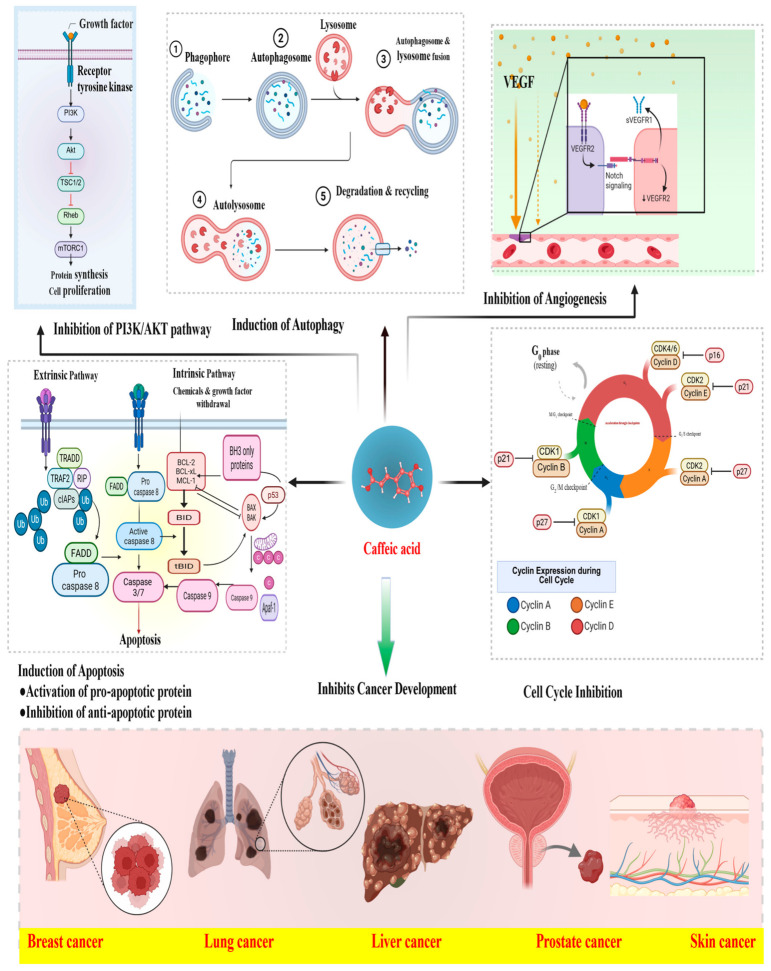
Caffeic acid inhibits cancer development through multiple molecular pathways, including suppression of the PI3K/AKT/mTOR signaling pathway, induction of autophagy, inhibition of angiogenesis, promotion of apoptosis, and regulation of cell cycle progression. Additionally, caffeic acid exerts anti-proliferative effects across various cancer types, including breast, lung, liver, prostate, and skin cancers. Additionally, CA induces apoptosis and cell cycle arrest. The figure was created using BioRender, accessed on 29 December 2025 https://app.biorender.com.

Cancer is a multifactorial disease, with its incidence increasing worldwide. Current treatment modes, including surgery, chemotherapy, and radiation, often come with expensive and adverse effects, prompting the exploration of alternative therapeutic approaches. Caffeic acid plays a role in cancer management via inhibition of angiogenesis, induction of apoptosis, and inhibition of cell proliferation (Figure 4 and Table 4). The study of caffeic acid offers promising avenues for developing safer and more effective cancer treatments. Its role in the management of different malignancies is described here.

#### 4.5.1. Lung Cancer

Lung malignancy is the leading cause of death, and incidence is growing globally. Existing treatment choices, for example, surgery, chemotherapy, and radiation, frequently carry high costs and side effects, leading to a search for alternative therapeutic methods. Studies have confirmed caffeic acid’s role in lung cancer. Caffeic acid is recognized as an inhibitor of TMEM16A through fluorescence quenching and whole-cell patch-clamp experiments. It regulated the proliferation, migration, and apoptosis of lung cancer cells by targeting TMEM16A. Western blot analysis revealed that caffeic acid regulates lung cancer growth by modulation of the MAPK signaling pathway. Moreover, in vivo tumor xenograft experiments exhibited that a combination of 5.4 mg/kg of caffeic acid as well as 4.1 mg/kg of DOX achieved an 85.6% tumor suppression rate [73]. A combination therapy using low-dose caffeic acid (CA) as well as paclitaxel (PTX) was found to inhibit the proliferation of NSCLC H1299 cells, whereas normal Beas-2b cells remained unaffected. Flow cytometry analysis revealed an increase in apoptosis in H1299 cells, which also showed sub-G1 phase arrest following CA treatment. Additionally, CA exposure led to elevated activities of Caspase-3 as well as Caspase-9. In vivo studies using H1299 xenografts established that the dual treatment of PTX and CA more efficiently suppressed tumor growth, without inducing significant adverse effects [74]. Another study examined the role of CA in lung cancer and its effects on cancer cell proliferation. Growth assays revealed that CA moderately promoted the proliferation of lung cancer cells. Moreover, pretreatment with CA attenuated the antiproliferative effects of a sub-IC_50_ concentration of paclitaxel, a commonly used chemotherapeutic agent. CA upregulated the expression of the pro-survival proteins Bcl-2 and survivin, which are regulated downstream of NF-κB [75].

#### 4.5.2. Breast Cancer

Breast cancer represents a substantial global health issue, being the most diagnosed cancer around the world [76,77]. In 2020, there were around 2.26 million reported cases, making it the leading cause of cancer-related fatalities among women [78]. Although there have been improvements in breast cancer treatment, considerable disparities continue to exist in terms of screening, diagnosis, and management, particularly in semi-urban and rural regions where healthcare access is often restricted [79]. Natural compounds are considered important complementary and alternative therapies with a considerable role in cancer management. These compounds have been chief medications in numerous ancient civilizations and have been given substantial consideration in the field of cancer drug discovery. The impact of CA and gallic acid on apoptotic gene expression was assessed in a breast cancer cell line. Treatment by this compound influenced the expression levels of P21, Mcl-1, and P53 genes, through the activation of the intrinsic apoptotic signaling pathway. CA and gallic acid induce toxic activities and morphological changes in this cancer cells by apoptosis, advocating their role as future antitumor agents. Based on the findings, one study concluded that through induction of apoptosis, CA and gallic acid induced toxic activity as well as morphological changes, demonstrating probable application as anti-tumor agents [80].

Caffeic acid (CA) inhibited the growth of triple-negative breast cancer (TNBC) cells and reduced the size of cancer stem cell-derived spheres. Additionally, it lowered reactive oxygen species (ROS) levels and caused mitochondrial membrane potential disruption. CA also impacted the stem-like properties of TNBC cells through the stem cell marker CD44 downregulation [81]. The impact of CA on the growth of breast cancer cells was determined, in addition to determining the contributory role of caspases, oxidative status, and mitochondria. MDA-MB-468 and MCF-7 breast cancer cells were exposed to different concentrations of CA for different time periods. The results demonstrated that caffeic acid reduced the percentage of MDAMB-468 and MCF-7 cells in a way that depended on the dose and duration of exposure. The breast cancer cells’ death induced by caffeic acid was related to an ROS level increase in both cell lines. The reduction in mitochondrial membrane potential by treatment with CA proposes that mitochondrial dysfunction may be involved in breast cancer cell death caused by caffeic acid. Prominently, caspase 8 activity increased by the treatment [82]. A breast cancer cells-based study demonstrated that cell migration analysis exhibited that caffeic acid (CA) and metformin significantly inhibited cancer cell migration, both alone and in combination. Colony formation examination revealed that CA totally inhibited colony formation. Overall, CA and metformin revealed the ability to inhibit cancer cell apoptosis, migration, modulation of tumor microenvironment, and metastasis [83].

#### 4.5.3. Cervix Cancer

Cervical cancer continues to be a major cause of death among women worldwide, impacting nearly 500,000 women each year [84]. While mortality rates have evidently dropped in high-income countries over recent decades, they remain noticeably elevated in low- and middle-income regions, which account for 88% of all deaths [85]. Caffeic acid is considered a promising complementary and alternative therapeutic agent with a significant role in cervical cancer management. It was reported that caffeic acid reduced proliferation of cervical cancer HeLa cells in a concentration-dependent way. Morphological indication of apoptosis, together with nuclear fragmentation, was noticed 24 and 48 h after treatment with caffeic acid. Time-dependent inhibition was also noticed [86].

A study based on human cervical cancer cell lines was conducted to evaluate the effects of Cisplatin (CDDP) as well as caffeic acid (CFC), both individually and in combination. Results exhibited that each compound alone inhibited the cervical cancer cells’ proliferation. Remarkably, the combination of CDDP and CFC further suppressed the growth of HeLa and CaSki cells. A combination index (CI) of less than 1 designated a synergistic effect between CDDP and CFC in these two cell lines [87]. Cervical cancer (SiHa) cells were treated with metformin (Met), caffeic acid (CA), or their combination. Both agents exhibited selective cytotoxicity toward cancer cells. Additionally, CA and Met modulated metabolic reprogramming in SiHa tumor cells. Additionally, CA and Met increased Cisplatin activity against quiescent tumor cells [88].

#### 4.5.4. Liver Cancer

Liver cancer ranks among the leading causes of cancer-associated mortalities globally and is classified into primary as well as secondary liver cancer [89]. A range of treatment choices are available for managing this disease. However, in spite of undergoing curative surgery, patients still face significant challenges, including metastasis, elevated recurrence rates, and a usually poor prognosis [90]. A study result demonstrated that CA but not ferulic, cinnamic, or sinapic acids inhibited proliferation of HCC cells, and cell numbers reduced through inducing apoptosis. Only transient CA exposure is required for these lethal activities that are linked with mitochondrial membrane potential disruption and induction of ROS. These outcomes support the use of CA as an anti-tumor agent to inhibit this cancer [91].

One study was conducted to develop anti-cancer drugs with precise tumor regression and anti-metastatic effect, having inhibitory activities of specific MMP-2,-9 enzyme activities and gene transcription. CA as well as CAPE selectively inhibited MMP-2 as well as -9. Treatment of HepG2 cells by CA and CAPE suppressed phorbol 12-myristate 13-acetate (PMA)-induced expression of MMP-9 through inhibiting the function of NF-κB. Correspondingly, CA as well as CAPE suppressed the growth of tumors and oral and subcutaneous CA and CAPE administrations reduced the liver metastasis. The outcomes confirm the therapeutic role of these compounds, and the anti-tumor and anti-metastatic potential of CA are driven by the selective inhibition of MMP-9 enzyme activity, along with transcriptional downregulation by dual suppression of MMP-9 and NF-κB catalytic activity [92].

#### 4.5.5. Gastric Cancer

Gastric cancer (GC) continues to be one of the most common and lethal cancers around the world, holding the position of the fourth leading cause of cancer-related mortalities worldwide [93,94]. Although improvements in diagnostic methods as well as therapies have increased survival in high-income countries, gastric cancer (GC) continues to affect particular racial and ethnic populations [95]. Moreover, current treatment strategies are often associated with adverse side effects. Therefore, there is an urgent need to develop cost-effective and efficient anticancer agents to overcome the restrictions of current therapies. A study based on gastric cancer reported that in SCM1 human gastric cancer cells, caffeic acid-induced [Ca2+] *i* increases through evoking phospholipase C-dependent Ca2+ release from the endoplasmic reticulum as well as Ca2+ entry through store-operated Ca2+ channels [96].

#### 4.5.6. Renal Cancer

A research study was conducted to measure the anticancer effects of CA and its derivative CADPE, focusing on their ability to target STAT3. The findings showed that both CA and CADPE expressively inhibit STAT3 activity, which then leads to a reduction in HIF-1α activity. In experiments with mice that had Caki-I carcinoma, administration of either CA and CADPE led to a decrease in tumor growth, as well as suppression of STAT3 phosphorylation, HIF-1α expression, and overall tumor vascularization. Taken together, the findings demonstrate that CA as well as CADPE act as potential STAT3 inhibitors and suppress tumor angiogenesis through blocking STAT3 activity and reducing the expression of HIF-1α and VEGF [97].

#### 4.5.7. Oral Cancer

Oral cancer is one of the furthermost common malignancies among the head and neck cancer group, accounting for around 389,485 new cases and 188,230 deaths each year [98,99]. Globally, it is the sixteenth most diagnosed cancer. The disease shows a remarkably high prevalence in Southeast Asia, where it has the highest incidence among men and ranks as the third most commonly detected cancer in women [98,100]. A study was conducted to assess the effect of caffeic acid (CA) on the viability as well as migration of malignant oral epithelial keratinocytes. It was demonstrated that very low concentrations of ethanol ranging between 2.5 and 10 mmol/L induce the oral squamous cell carcinoma cells’ viability, whereas the addition of caffeic acid shows an antagonistic activity, reducing pro-proliferative ethanol activity. Moreover, the biological activity of caffeic acid suppressed the migratory capacity of oral squamous carcinoma cells [101]. In human squamous cell carcinoma cells, the treatment of caffeic acid at a concentration of 65 μg/mL resulted in decreased cell viability as well as an upregulation of p53 protein expression. This protein is vital for promoting cell cycle arrest and apoptosis [102]. The apoptotic effects of caffeic acid (CA) and its derivative caffeic acid phenethyl ester (CAPE) on apoptosis and cell proliferation in human head and neck squamous carcinoma cells line (Detroit 562) were examined. It was reported that exposure to CAPE and CA was found to result in a dose-dependent reduction in the viability of cancer cells at different levels. Treatment by CA/CAPE suggestively affect the cells’ viability. It was further demonstrated that treatment of Detroit 562 cells with CA and CAPE induced apoptosis at concentrations of 50 μM as well as 100 μM. At the higher dose (100 μM), there was a prominent increase mainly in late-stage apoptosis. Moreover, CA and CAPE treatment influenced the distribution of cells in the G0/G1 phase [103].

#### 4.5.8. Osteosarcoma

An osteosarcoma-based study reported that MG63 cells were significantly induced to undergo apoptosis by caffeic acid (10 µg/mL). The apoptotic phenomenon caused by caffeic acid and the inhibition of Z-VAD-FMK were established by DAPI staining as well as TUNEL assay. Cleaved caspase-8, -9 and -3 were formed apparently upon the caffeic acid treatment. Pretreatment of Z-VAD-FMK inhibited the cleaved caspase-8, -9, and -3 [104]. A study examined the involvement of cytochrome c (Cyt c) release as well as the activation of BH3-interacting death (Bid) in caffeic-acid-induced apoptosis in MG-63 osteosarcoma cells. The outcomes exhibited that mitochondrial levels of Cyt c and Bid slowly decreased over time, whereas the amounts of truncated Bid (t-Bid) as well as cytosolic Cyt c increased in a time-dependent manner. Moreover, in MG-63 cells pretreated with Z-VAD-FMK (100 μM), mitochondrial Cyt c and Bid levels were significantly elevated. In contrast, the concentrations of t-Bid as well as cytosolic Cyt c were decidedly reduced after CA treatment compared with cells that did not receive the pretreatment [105].

Another study investigated the effects of caffeic acid on PKCδ translocation and mitochondrial membrane potential (ΔΨm) in MG-63 cells. The finding concluded that caffeic acid induced apoptosis in MG-63 osteosarcoma cells by promoting the translocation of PKCδ to the mitochondria and reducing mitochondrial membrane potential, which might cause MMP [106]. Another study found that in pre-treated MG-63 cells-induced mice, the volumes of the tumor masses decreased in a dose-dependent manner with the administration of caffeic acid. Specifically, a pre-treatment with 10 µg/mL of caffeic acid significantly reduced the mass formation [107].

#### 4.5.9. Leukemia

A study focused on chronic myeloid leukemia revealed that the decrease in cell proliferation following caffeic acid (CA) treatment was associated with an increased expression of two cell cycle repressor genes, CHES1 and CDKN1A. Additionally, CA treatment induced apoptosis and influenced proliferation in IM-resistant cells. Finally, the combined treatment of IM and CA at suboptimal concentrations demonstrated a synergistic effect in inhibiting cell proliferation and inducing apoptosis. The ability of CA to enhance the anti-leukemic activity of IM underscores its potential as a nutraceutical agent in the management of CML [108].

#### 4.5.10. Prostate Cancer

Prostate cancer (PC) ranks sixth among male cancer-related deaths globally and is the most diagnosed malignancy in men [109]. The existing treatment approaches lead to adverse side effects. However, there is a pressing need for affordable and effective anticancer drugs to tackle the challenges posed by current treatment options.

The role of CA in inhibiting IL-6/JAK/STAT3-mediated proliferative signaling was evaluated in human prostate cancer cells. CA treatment meaningfully suppressed the proliferation of PC-3 and LNCaP cells in a concentration-dependent manner and promoted ROS generation, apoptosis, and cell cycle arrest. Additionally, CA reduced the phosphorylation form of MAPK families, including ERK1, JNK, and p38, in PC-3 cells. Apoptotic induction by CA was additionally supported by the downregulation of the anti-apoptotic protein Bcl-2 and upregulation of Bax and caspase-3 expression prostate cancer cells [110]. Another study was done to explore the role of CA on the proliferation, migration, and stem cell-like properties of prostate cancer cells DU-145. It was reported that CA reduced cell proliferation in a dose- as well as time-dependent way without effect on cell cycle progression. Additionally, CA reduced the cancer stem cell population and inhibited stem cell-like properties. These results advocate for CA to be considered in the development of improved chemotherapy against this cancer [111].

#### 4.5.11. Skin Cancer

The protective effects of caffeic acid (CA) against inflammation as well as photocarcinogenesis caused by both acute and chronic UVB radiation were examined in a mouse model. UVB exposure for 30 weeks caused the development of squamous cell carcinoma in the animals. This prolonged irradiation was also associated with downregulation of p53 and increased expression of iNOS, VEGF, and TGF-β, along with a higher incidence of skin tumors. However, administration of caffeic acid through topical application or intraperitoneal injection prior to each UVB exposure significantly increased p53 expression, while suppressing iNOS, VEGF, and TGF-β levels, thereby lowering tumor multiplicity in the skin [112]. The study results reported that treatment with CA in melanoma cells resulted in the induction of apoptosis, reduced cell viability, and an increase in p-GSK3β levels. In addition, CA treatment promoted the upregulation of p21 and p53, whereas the expression of cyclin D1 and the anti-apoptotic protein Bcl-2 was significantly reduced [113].

**Table 4:** This table presents a summary of preclinical evidence regarding the anticancer action of caffeic acid (CA) through modulation of cell signaling pathways, including induction of apoptosis and sub-G1 cell cycle arrest, anti-cell proliferation, reduction of cancer cell viability, and suppression of tumor angiogenesis.

Cancer	Study Types	Model/Cell Line	Key Findings	Refs.
Lung cancer	In vitro	H1299 cells	° CA induces apoptosis and sub-G1 cell cycle arrest ° CA activate caspase-3 and caspase-9	[74]
	In vivo	H1299 xenografts in nude mice	° CA treatment retarded the growth	[74]
	In vitro	A549 cells	° CA rescues the PTX-induced anti-proliferation	[75]
Breast cancer	In vitro	MDA-MB-231 and MDA-MB-468 cells	° CA-induced apoptosis and block in the G2/M phase	[81]
	In vivo	TNBC xenografts in mice	° CA inhibits growth and improves the tumor microenvironment	[81]
	In vitro	MCF-7 and MDA-MB-468 cells	° CA reduced cancer cell viability	[82]
Cervix cancer	In vitro	HeLa cells	° CA induces apoptosis	[86]
	In vitro	HeLa, CaSki, SiHa and C33A cells	° Growth of cancer cells prevented by CA	[87]
Liver cancer	In vitro	HepG2 cells	° CA decreases MMP-9 enzyme activity and MMP-9 expression	[92]
	In vivo	HepG2 tumor xenografts in nude mice	° Reduction of tumor size CA-treated group ° Decreased metastatic foci number in the liver	[92]
Gastric cancer	In vitro	SCM1 cells	° CA inhibited cell viability and induced apoptosis	[96]
Kidney cancer	In vitro	Caki-I cells	° CA is inhibitors of STAT3 ° CA suppresses tumor angiogenesis	[97]
	In vivo	Caki-I carcinomas in nude mice	° CA retarded tumor growth	[97]
Head and neck cancer	In vitro	Detroit 562 cells	° CA decreases the viability of cancer cells ° CA induced apoptosis	[103]
Bone cancer	In vitro	MG63 cells	° Apoptosis induced by CA	[104]
	In vitro	MG 63 cells	° CA promotes the release of Cyt c by activating Bid	[105]
	In vitro	MG63 cells	° CA triggers apoptosis	[106]
Leukemia	In vitro	K562 cells	° CA induced apoptosis and caused anti-proliferative effect ° Administration of IM and CA showed a synergy of action	[108]
Prostate cancer	In vitro	PC-3 and LNCaP cells	° CA inhibits cancer cells proliferation, cell cycle arrest	[110]
	In vitro	DU-145 cells	° CA decreased cell proliferation and inhibited cell migration	[111]
Skin cancer	In vitro	G361 and SK-MEL-24 cells	° CA exposure decreased cell viability, triggered apoptosis	[113]

### 4.6. Cardioprotective Effects

Caffeic acid exhibits cardioprotective effects through various mechanisms. Its roles in providing protection to the heart are indicated by cardiomyocyte protection. By targeting these pathways, caffeic acid reveals its role as a protective agent for cardiovascular health. The pharmacological activities of caffeic acid as cardioprotective are noted by modulating oxidative stress, inflammation, and maintenance of tissue structure (Table 5).

It was reported that oral administration of captopril, chlorogenic acid, and caffeic acid normalized hypertensive activity caused by administration of cyclosporine. CA and chlorogenic acid diminished heart rates (HR), systolic blood pressure, activity of BChE ACE, and AChE, and arginase in the treated hypertensive rats [114].

The study was designed to explore whether CA exerts a cardioprotective role to inhibit myocardial fibrosis post-myocardial infarction (MI). Histological investigations demonstrated that CA ameliorated ventricular remodeling and partially restored cardiac function. This compound selectively targeted TGFBR1 and collagen deposition reduction, and inhibited TGFBR1-Smad2/3 signaling. Additionally, CA dose-dependently reduced the collagen synthesis, proliferation, as well as TGFBR1-Smad2/3 pathway activation in primary cardiac fibroblasts stimulated by TGF-β1 in vitro. These results suggest that CA efficiently lessens myocardial fibrosis and improves cardiac function following MI [115]. The study assessed the preventive effect of caffeic acid on lipid peroxides, cardiac marker enzymes, antioxidants, and histopathological findings in rats with isoproterenol (ISO)-induced myocardial infarction. Oral pretreatment with caffeic acid led to a substantial reduction in serum levels of cardiac marker enzymes, plasma uric acid, and heart lipid peroxidation products, along with a noteworthy increase in the levels of antioxidants in the system. Histopathological examination of the myocardium further confirmed the protective role of CA in rats with myocardial infarction [116]. Bassim et al. (2014) performed a study to examine the cardioprotective activity of caffeic acid against doxorubicin-induced cardiotoxicity. Rats in the Dox + caffeic acid group showed reduced cytokine expression, improved LV function, decreased myocardial marker injury, and lower MDA and hs-CRP levels compared to the Dox group. The pathological outcome appeared approximately normal in Dox + caffeic acid without fibrosis [117].

### 4.7. Role of Caffeic Acid in Liver-Associated Pathological Conditions

**Figure 5:**
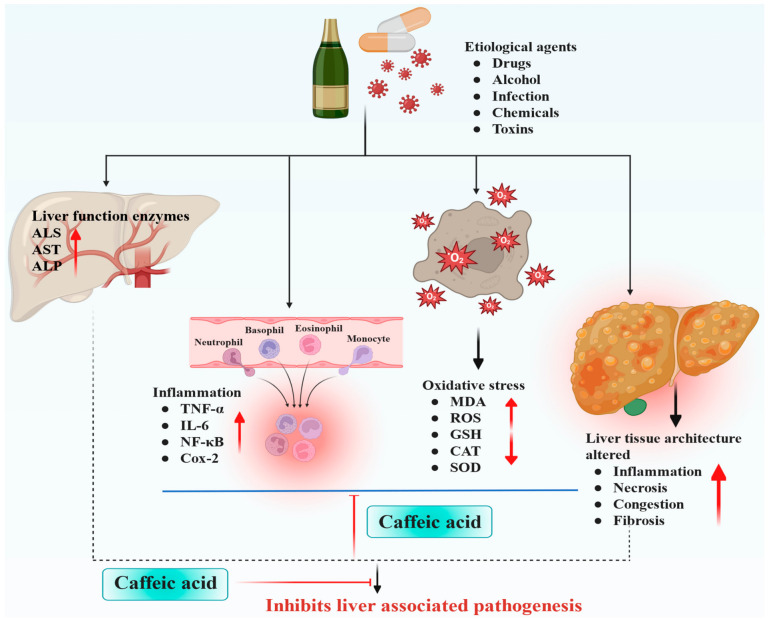
Hepatoprotective effects of caffeic acid against liver-associated pathological conditions induced by various etiological agents. These harmful stimuli elevate liver function enzymes, activate inflammatory responses, and promote oxidative stress characterized by increased MDA and ROS with reduced antioxidant defenses. These pathological changes ultimately disrupt liver tissue architecture. Caffeic acid mitigates these adverse effects by suppressing oxidative stress and inflammation, in that way protecting liver tissue integrity. The downregulation is indicated by the downward-directing arrow, while the upregulation is denoted by the upward arrow. The figure was created using BioRender. Rahmani AH. (2026). https://app.biorender.com, accessed on 29 December 2025.

Caffeic acid shows a promising role in the management of liver disease through the regulation of inflammation (inhibition of proinflammatory cytokines), improvement of antioxidant enzymes (SOD, CAT, and GSH), reduction of oxidative stress (inhibition of MDA and ROS), and maintenance of liver tissue architecture, as presented in Figure 5. Moreover, the hepatoprotective activity of caffeic acid is reported through different mechanisms, as discussed in various studies and summarized in Table 5. One study aimed to examine the role of caffeic acid (CA) on metabolically associated steatotic liver disease (MASLD). CA significantly improved liver damage, inflammatory injury, and steatosis and raised the NAFLD activity score (NAS), which was reduced in HFD-fed mice [118].

Another study intended to explore the effects of CA treatment on gut microbiota composition as well as metabolic functions in a mouse model of nonalcoholic fatty liver disease. Mice fed a high-fat diet exhibited obesity, higher serum biochemical parameters, increased intrahepatic lipid accumulation, and altered gene expression related to lipid metabolism. Treatment with CA reverted the gut microbiota imbalance and mitigated lipopolysaccharide-driven inflammation, therefore preventing the deregulation of lipid metabolism-related gene expression [119].

The effect of CA on hepatic steatosis and its mechanism of action was evaluated. CA (50 µM) treatment in palmitate-treated AML12 hepatocytes decreased ER stress, reduced lipid accumulation and lipogenesis markers, and increased autophagy markers. CA dropped liver and body weights. Lipid accumulation in the liver was reduced in the HFD + CA group as compared to the HFD group [120]. The experiment explored the hepatoprotective role of caffeic acid (CA), rosmarinic acid (RA), and their combined administration. Oral intubation of CA or RA alone for five consecutive days prior to tert-butyl hydroperoxide exposure reduced markers of hepatic toxicity, including alanine aminotransferase, aspartate aminotransferase, lipid peroxidation, oxidized glutathione, and enzyme activities related to antioxidants. Notably, the combined treatment of CA and RA produced a greater reduction in lipid peroxidation and a more pronounced increase in hepatic endogenous antioxidant enzymes and glutathione (GSH) levels compared with either compound administered alone [121]. This study was performed to evaluate the protective effect of caffeic acid on hepatic and renal injury caused by capecitabine administration. Treatment by CA reduced the elevated plasma biomarkers related to liver and kidney injury and improved both enzymatic as well as non-enzymatic antioxidant levels in hepatic tissue. Based on the findings, the study concluded that the protective role of caffeic acid may be attributed to its ability to improve the antioxidant defense system by reducing lipid peroxidation [122]. The protective effects of CA on the liver as well as kidneys were checked in mice exposed to arsenic. Results showed that arsenic exposure elevated levels of AST, ALT, LDH, creatinine, and urea. However, when CA was administered alongside arsenic, there was a notable decrease in serum AST and creatinine levels. Moreover, liver regeneration and renal glomeruli in the mice that received CA demonstrated its protective effects on both organs. Histopathological changes caused by arsenic, such as degeneration, tissue hypotrophy, hyperemia, and necrosis in liver and kidney tissues, were noticed to revert to normal after the administration of CA [123].

### 4.8. Anti-Colitis Effects

The anti-colitis effects of caffeic acid (CA) arise from multiple mechanisms that work at various levels in the body. CA helps reduce inflammation and oxidative stress, supports gut microbiota balance, and promotes mucosal healing, together contributing to enhanced intestinal health (Table 5). Additionally, CA plays a role in maintaining the colon tissue architecture through the reduction/control of histopathological changes.

The protective role of caffeic acid against dextran sulfate sodium (DSS)-induced intestinal injury in *Drosophila melanogaster* model was investigated. CA supplementation significantly improved intestinal damage in UC flies by restoring excretion balance, improving acid-base balance, repairing intestinal atrophy, inhibiting intestinal structural destruction, reducing the number of harmful bacteria, inhibiting intestinal epithelial cell death, and inhibiting excessive intestinal stem cell proliferation [124].

This study examined the role of caffeic acid (CA) supplementation in a mouse model of dextran sulfate sodium-induced colitis. CA shows anti-inflammatory activity, and it suppresses the secretion of IL-6, IFNγ, and TNFα and the colonic infiltration of CD3^+^ T cells, F4/80^+^ macrophages, and CD177^+^ neutrophils through the activation of NF-κB signaling pathway inhibition. Examination of fecal microbiota exhibited that CA restores the decrease in richness as well as inhibiting the increase in the ratio of *Firmicute* to *Bacteroidetes*. CA intensely enhances the proportion of the mucin-degrading bacterium *Akkermansia* in colitis mice [125].

The study results confirmed that CA evidently alleviates mucosal inflammation. This was evidenced by improvements in disease severity, serum biochemical parameters, preservation of epithelial and crypt architecture, reduction of mucosal ulceration, and decreased secretion of inflammatory cytokines in colonic homogenates and explant cultures. Consistently, CA limited both the infiltration and functional activity of mononuclear macrophages in the colonic mucosa, MLNs, and spleen in ulcerative colitis (UC). Moreover, CA directly suppressed the activation of BMDMs following stimulation with TLR agonists in vitro. Overall, CA attenuated DSS-induced murine UC by inhibiting macrophage activation, suggesting its role as an alternative therapeutic strategy for UC [126]. One study assessed the role of caffeic acid (CA) in regulating pathways involved in intestinal inflammation. The results showed that CA (i) modulates COX-2 activity, its product PGE_2_, and IL-8 production in IL-1β-stimulated cells; (ii) inhibits the formation of AGEs; and (iii) demonstrates strong chelating activity [48]. A study was performed to check whether CA has a protective effect on colonic inflammation. A 251 mg/kg supplementation of CA was to given colitis mice. The results showed that CA treatment recovered DSS-induced disease activity index (DAI), colon length, and histopathology scores of colon tissue. Furthermore, CA treatment meaningfully increased the level of IL-10, total antioxidant capacity, SOD, GSH-Px, and CAT and reduced pro-inflammatory cytokines and MDA levels. Moreover, supplementation of CA prevented gut barrier injury through *Occludin* gene expression enhancement. Additionally, it altered the gut microbiome composition through enhancing the relative abundance of *Alistipes* and *Dubosiella* and reducing the relative abundance of *Bacteroides* and *Turicibacter* [49].

### 4.9. Role of Caffeic Acid in Reproductive System

**Figure 6:**
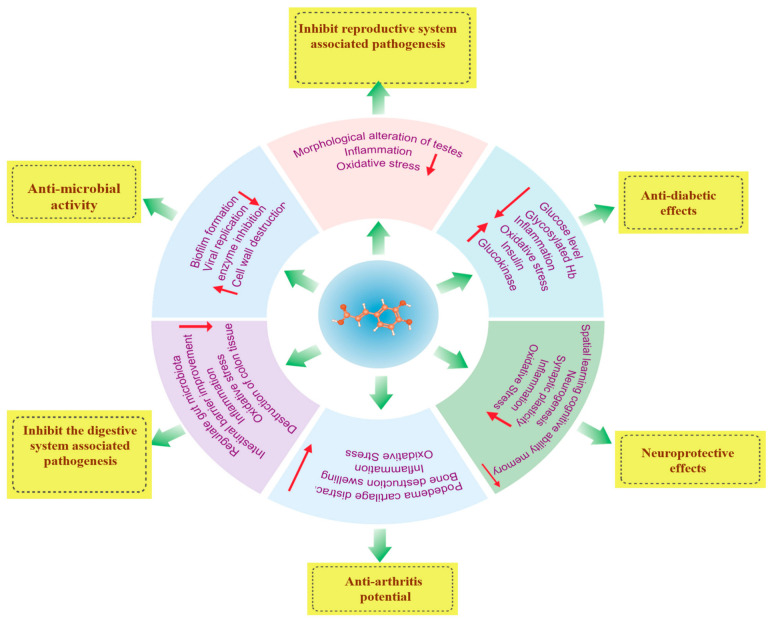
Caffeic acid exhibits protective activities against multiple pathological conditions, including reproductive system disorders, diabetes, neurodegenerative diseases, arthritis, digestive system disorders, and microbial infections. The compound exhibits antimicrobial activity by inhibiting biofilm formation, and microbial cell wall integrity. It inhibits various diseases by reducing inflammation, alleviating oxidative damage, and protecting tissue architecture. In addition, its role in diabetes is noted as regulation of blood glucose, enhancement of insulin, and maintenance of tissue structure. These multifunctional properties highlight the wide-ranging therapeutic effect of caffeic acid in the management of various diseases. The downregulation is indicated by the downward-directing arrow, while the upregulation is denoted by the upward arrow. The figure was created using BioRender accessed on 29 December 2025. Rahmani AH. (2026). https://app.biorender.com.

Caffeic acid contributes to the management of reproductive system–related pathologies by multiple mechanisms (Figure 6). Caffeic acid modulates key cellular signaling pathways and supports hormonal balance. By addressing these pathways, caffeic acid shows promise in managing reproductive system-related diseases. The study evaluated the effects of caffeine combined with CA on some biomarkers of male reproductive function in rats. The outcomes revealed substantial increase in reproductive hormone, epididymal and testicular nitric oxide levels of the rats. Furthermore, reduced oxidative stress in the testes as well as epididymides of the treated rats. Likewise, reduced testicular cholesterol level with associated raise in testicular steroidogenic enzyme activities, zinc and glycogen levels were detected in the treated rats. This study proposes the combination therapy of caffeine as well as CA at the dose tested for improving reproductive function [127]. A recent study was made to assess the protective activity of caffeic acid on basal human semen and under induced oxidative stress (OS). It was reported that caffeic acid exhibited protective effects on sperm damage induced by H_2_O_2_ treatment, restoring motility, acrosome status and DNA integrity, and decreasing F_2_-isoprostane levels. Expression HO-1 and Nrf2 were upregulated by CA downregulated by H_2_O_2_, as well as restored by the co-treatment. Supplementation of CA protects human spermatozoa during in vitro handling through OS reduction, improving sperm parameters [128].

A study was performed to examine the therapeutic role of caffeic acid on polycystic ovary syndrome (PCOS). Ovarian granulosa cell line (KGN cells) was treated with H_2_O_2_ to induce oxidative stress, and the effects of caffeic acid on the protein expression of apoptosis-related markers were assessed. It was noticed that caffeic acid inhibits intracellular ROS generation as well as protecting the human ovarian granulosa cell line against oxidative stress. For the in vivo characteristic of the study, female rats were utilized to induce the PCOS model, Caffeic acid efficiently improved irregular estrous cycles in DHEA-induced PCOS rats. The results suggest that caffeic acid has a promising role in decreasing oxidative stress-induced damage as well as ameliorating PCOS-associated complications through modulating ER stress [129].

A study was done to examine the protective role of caffeic acid against acrylamide-induced reproductive dysfunction in rats. It was noticed that acrylamide causes substantial alteration in serum concentrations of testosterone, FSH, and LH, as well as reduced sperm motility and viability. Furthermore, acrylamide causes histological changes and promotes DNA damage. After treatment of caffeic acid, serological, biochemical, and histological changes returned to nearly normal ranges [130]. Another study was performed to estimate the protective effect of CA against arsenic (As)-induced testicular damage in mice. The results showed that arsenic exposure suggestively decreased testicular FRAP, GPx, and SOD activities, along with reducing plasma levels of testosterone and dihydrotestosterone compared with the control group. Moreover, arsenic caused noteworthy histopathological and morphological changes in the testes. However, co-administration of CA with arsenic improved antioxidant parameters, restored testosterone and dihydrotestosterone levels, reduced MDA levels, and alleviated the observed histopathological injury [18].

The effect of CA treatment on ectopic and eutopic endometrial cells’ enzyme activities was examined. In ectopic endometrial cells, CA caused an important elevation in Nrf-2 gene expression level, HO-1 and NQO1, and enzyme activities. Furthermore, ROS level reduction was determined in CA-treated ectopic endometrial cells. CA protects the endometrial cells and is capable of preventing the progression of endometriosis as well as its associated complications [131].

The study examined the protective role of CA against oxidative stress, inflammation, and apoptosis induced by aflatoxin B1 (AFB1) in the hypothalamus, testis, and epididymis of rats. The results indicate that the toxic effects of AFB1 on biochemical markers in these tissues were significantly alleviated in rats treated with CA alongside AFB1. Co-treatment with CA also mitigated the reduction in antioxidant levels and limited the increase in lipid peroxidation (LPO) and RONS. Furthermore, the AFB1-induced elevation of nitric oxide (NO), TNF-α, Bax expression, and MPO activity was markedly decreased in the hypothalamus, testis, and epididymis of CA-treated rats. Histological examination confirmed that CA reduced the severity of AFB1-induced tissue damage in these organs [132].

### 4.10. Anti-Obesity Effects

Caffeic acid exhibits anti-obesity effects by several mechanisms. It can regulate lipid metabolism through inhibiting fat accumulation as well as promoting lipolysis. Additionally, caffeic acid affects key signaling pathways involved in obesity and associated complications. Through the modulation of different activities, this compound shows its role as an anti-obesity agent. The anti-obesity activity of caffeic acid (CA) and chlorogenic acid (CGA) as co-treatment in human adipocytes was determined. It was reported that CA/CGA combination induced lipolysis and upregulated browning gene expression [133]. A study was done to check the effect of caffeic acid (CA) on the reduction of intracellular lipid accumulation, ROS formation, and mitochondrial transmembrane potential changes in differentiated 3T3-L1 cells. CA causes lipid content reduction in the cells submitted to the post- and co-treatment. Caffeic acid caused reduction in the formation of intracytoplasmic ROS. The treatment with CA protects against oxidative stress caused in mitochondria. Thus, CA acts on adipogenesis, dropping intracellular accumulation of lipids in the 3T3-L1 cells [134]. The role of three compounds such as astaxanthin (ATX), hydroxytyrosol (HT), and caffeic acid (CA) on zebrafish (Danio rerio) larval adiposity as well as rainbow trout (Onchorynchus mykiss) adipocytes was evaluated. The zebrafish obesogenic test (ZOT) confirmed the anti-obesogenic effects of HT and CA. These compounds were able to counteract the obesogenic effect and suppressed lipid accumulation [135].

A study examined the effects of CA combined with arabinoxylan or β-glucan on glucose and lipid metabolism, gut microbiota, and metabolites in mice fed a high-fat diet (HFD). The combination significantly reduced blood glucose levels, body weight, and serum free fatty acid concentrations. Specifically, the combination of CA and β-glucan efficiently decreased hepatic lipid accumulation and serum total cholesterol levels. It also modulated inflammation and oxidative stress while improving gut barrier function. Furthermore, this combination reversed HFD-induced alterations in microbiota-derived metabolites [136]. This study was designed to examine the anti-obesity mechanism of CA and its association with its anti-obesity activity and alterations in gut microbiota in high-fat diet-induced obese (DIO) mice. The DIO mice were administered caffeic acid at a dose of 50 mg/kg body weight. The results confirmed that CA suggestively lessened obesity. In particular, CA reduced body weight, fat accumulation, and weight gain, enhanced energy expenditure, and improved lipid profiles in DIO mice. Additionally, CA helped restore gut microbiota imbalance and increased the abundance of anti-obesity-associated bacteria as well as butyrate-producing bacteria [137].

A study examined the effect of caffeic acid on obesity in mice that were fed a high-fat diet. Supplementation with caffeic acid lowered body weight, plasma GPT and GOT levels, visceral fat mass, FAS activity, and free fatty acids compared with the HFD group. Caffeic acid also dropped cholesterol and triglyceride concentrations in plasma and livers. Supplementation of caffeic acid suppressed the lipogenesis activity through sterol regulatory element-binding protein 1c as well as its target enzyme fatty acid synthase [138]. The study investigated the anti-obesogenic effects of CA or resveratrol and their effect on lipogenic enzymes. Based on the results, the study proposed that CA and resveratrol show regulatory activity concerning obesity-linked metabolic disorders, likely thru modulating lipogenesis as well as lipolysis-related proteins [139].

**Table 5:** This table summarizes the diverse pharmacological activities of caffeic acid as cardioprotective, hepatoprotective, anti-colitis, reproductive protective, anti-obesity, and anti-arthritic effects. The key findings demonstrate that caffeic acid employs protecting actions by modulating inflammation, enzymatic activity, and tissue damage.

Activity	Study Model	Dose	Key Findings	Refs.
Cardioprotective effect	Cyclosporine induced hypertensive rats	10 and 15 mg/kg	° CA lowered blood pressure. ° CA reduced the activities of important enzymes involved in the development of hypertension	[114]
	ISO-induced myocardial-infarcted rats	15 mg/kg	° CA ameliorates cardiac damage	[116]
	Doxorubicin induced cardiotoxicity rats	40 mg/kg	° CA reduced cytokine expression, decreased myocardial marker injury	[117]
Hepatoprotective effect	Oxidative stress-induced liver damage	100 mg/kg	° CA ameliorated the elevated plasma biomarkers ° CA improved antioxidant levels	[123]
Anti-colitis effect	DSS-induced colitis mice	50 mg/kg	° CA attenuates the mucosal inflammation	[126]
	Colitis caused by DSS in mice	251 mg/kg	° CA treatment recovered colon length ° Histopathology changes in colon tissue reduced by CA	[49]
Role in reproductive system	Acrylamide-induced male reproductive dysfunction rats	10, 20, 30, 40 mg/kg	° Histological and biochemical changes improved after CA	[130]
	Arsenic-induced testicular injury in mice	60 mg/kg body	° CA plus As attenuated histopathological alterations	[18]
	Aflatoxin B1-mediated toxicity in the rat reproductive system	20 or 40 mg/kg	° CA improved functional characteristics of spermatozoa	[132]
Role in digestive system	High fat diet induced obese mice model	50 mg/kg	° CA ameliorated obesity and fat accumulation	[137]
Anti-arthritis effect	Adjuvant-induced arthritis in rats	50 mg/kg/day	° Caffeic acid attenuated the severity of arthritis ° It mitigated paw edema and protected the joint tissues	[140]
	Adjuvant-induced arthritis rats	5, 25, 125 mg	° Caffeic acid decreased swelling in ankle joint	[141]

### 4.11. Anti-Arthritis Effects

Anti-arthritic effects of caffeic acid are described by its role in decreasing paw swelling, preservation of joint structure, and reducing damage to cartilage and bone tissues.

In a study, rheumatoid arthritis-derived fibroblast-like synoviocytes (RA-FLS) were treated with caffeic acid (CA) to investigate its effect on cytokine production, as well as the expression of MMP-1 and PGE_2_. The results indicated that elevated levels of CA induced apoptosis in RA-FLS. Furthermore, CA significantly reduced the production of pro-inflammatory cytokines IL-6 and TNF-α in these cells. Additionally, CA significantly suppressed the levels of MMP-1 and PGE_2_, both of which are crucial in the development of rheumatoid arthritis, at both transcriptional and translational levels. The study also showed that CA alleviates the inflammatory response in RA-FLS [47].

A study was conducted to assess the anti-arthritic properties of CA as well as ellagic acid using an adjuvant-induced arthritis model. Treatment with both compounds decidedly decreased paw swelling (edema) and limited the infiltration of inflammatory cells. Additionally, they helped preserve joint structure by preventing pannus development and reducing damage to cartilage and bone tissues. Furthermore, both compounds reduced the concentrations of key tissue-remodeling factors, including MMP-9 and VEGF, in the paws of arthritic rats. They also reduced serum oxidative stress as well as nitric oxide levels, whereby increasing reduced glutathione in the arthritic animals [140]. The role of caffeic acid in reducing inflammation as well as inhibiting osteoclastogenesis in adjuvant-induced arthritis (AIA) rats was examined. The ankle joints of AIA rats showed severe swelling prior to treatment. However, administration of caffeic acid significantly reduced swelling in a concentration-dependent way. More bone loss was also noticed in the ankle joints of AIA rats, whereas treatment by CA 125 mg remarkably inhibited this bone loss [141].

### 4.12. Wound Healing Activity

Caffeic acid exhibits wound healing activity by modulating inflammation as well as oxidative stress. A study was conducted to assess the wound-healing activity of caffeic acid in skin-incised mice. Caffeic acid displayed substantial anti-inflammatory activity as well as wound healing in incised-wound tissue. Then again, it significantly stimulated collagen-like polymer synthesis in NIH 3T3 fibroblast cells, whereas it inhibited both silica-induced ROS generation and melittin-induced arachidonic acid release as well as PGE_2_ production in Raw 264.7 cells [142].

### 4.13. Anti-Microbial Activity

Phenolic compounds, including caffeic acid, show antimicrobial effects against bacteria, viruses, and fungi, through multiple mechanisms, such as disrupting microbial membranes, inhibiting nucleic acid synthesis, and reducing biofilm formation. The antimicrobial effects of caffeic acid are described in Figure 6. One of the primary actions is its ability to disrupt the integrity of bacterial cell membranes, leading to cell lysis and death. Additionally, caffeic acid interferes with critical metabolic pathways within microbial cells, hindering their growth and reproduction. Another significant mechanism is its capacity to inhibit biofilm formation, a protective layer that bacteria create to shield themselves from environmental stresses and antimicrobial agents. Table 6 summarizes the antimicrobial, antiviral, and antifungal activities of caffeic acid.

i. Antibacterial activity

A study assessed the inhibitory effect of CA on the efflux pumps of resistant strains of Staphylococcus aureus. Specifically, caffeic acid was found to inhibit the MrsA pumps in the RN-4220 strain and the NorA 1199B strain. Additionally, the docking model confirmed that caffeic acid showed greater efficiency, which is in line with the experimental results indicating its effectiveness [143]. Another study was done to check the inhibitory effect of caffeic acid against *tet*-efflux pump-mediated tetracycline-resistant *Streptococci* spp. Caffeic acid demonstrates promising inhibitory activity to target *tetR and tetM* [144]. Another study finding reported that caffeic acid (CA) confirmed variable antibacterial effects on *S. aureus* strains. Supplementation of Mueller–Hinton agar (MHA) with one-quarter of the MIC of CA enhanced the antibacterial activity of erythromycin, cefoxitin, and clindamycin, and, to a lesser extent, vancomycin. These findings suggest that CA possesses antibacterial activity against clinical *S. aureus* strains and may employ a synergistic antimicrobial effect when used in combination with antibiotics [145]. Tianle Xu et. al, 2022 reported that supplementation of CA inhibits the growth of the major strains of bacteria isolated from clinical bovine mastitis milk samples. CA was found to disturb the biofilm formation of *E. coli* B1. Moreover, the pretreatment of bovine mammary epithelial cells (bMECs) by CA restored changed lipid homeostasis produced by stimulation of *E. coli*. The role of CA was established through the CA administration in mice followed by Gram-negative bacterial infection. Together, these results show the ability of CA to mediate Gram-negative infections and demonstrate that it has the capability to be developed as an innovative antibacterial drug [146]. Another study was designed to assess the antimicrobial effect of CA against staphylococcal strains. The outcomes exhibited that CA inhibited the growth of all tested isolates, as well as reducing bacterial cell viability. When combined with erythromycin, CA showed a synergistic effect against three of the tested strains and had an additive effect against two others [147].

ii. Anti-viral activity

A study evaluated the antiviral activity of caffeic acid (CA). It’s in vitro effects on ILHV replication were evaluated in Vero and A549 cell lines, demonstrating that caffeic acid (500 µM) revealed virucidal activity against ILHV [24]. A comparison of the one-step growth curves of influenza virus replication, conducted with and without CA, revealed that the eclipse phase of viral multiplication in infected cells remained unaffected. However, the production of progeny viruses was suggestively reduced in the presence of CA. Further experiments showed that the antiviral effect of CA was most effective when added at an early post-infection stage. Along with the decrease in viral yield, there was also a significant reduction in virus-induced cytopathic effects and apoptotic nuclear fragmentation due to the presence of CA. This suggests that the compound suppresses the degeneration of virus-infected cells [148]. The duck hepatitis B virus (DHBV) infection model and HepG2.2.15 cell line was utilized as in vivo and in vitro models to evaluate their anti-HBV effects. In the cell model, all three compounds tested—CA, chlorogenic acid, and quinic acid—showed the ability to inhibit HBV-DNA replication and reduce HBsAg production. Moreover, both chlorogenic acid and CA resulted in decreased serum DHBV levels in the model involving DHBV-infected ducklings [149]. The antiviral activity of caffeic acid against canine distemper virus (CDV) was assessed through in vitro experiments. The results indicate that the half-maximal inhibitory concentration (IC_50_) of caffeic acid against CDV at 1 and 2 h post-infection (PI) was 23.3 μg/mL and 32.3 μg/mL, individually. Steadily, at 1 and 2 h PI, the caffeic acid exhibited a diminished (23.3–57.0% as well as 37.2–38.1%) viral inhibitory role in Vero cells. In addition, caffeic acid was found to suppress the overall synthesis of viral RNA. The data suggests that CA effectively inhibits CDV infection in Vero cells, representing its use in treating clinical diseases related to CDV infection [150]. The virological properties as well as antiviral effects of CA against HSV-1 were determined. CA was found to inhibit the replication of HSV-1 in vitro. The one-step growth curve showed that adding CA 8 h post-infection (h.p.i.) did not impact the production of progeny viruses. However, an analysis of the timing of CA addition revealed that introducing it early after infection reduced the formation of infectious progeny viruses [151].

iii. Antifungal activity

A study investigated the antifungal action of CA and nano-CA. As per the MIC_50_ and MIC_90_ values, nystatin, fluconazole carrier, nano-caffeic acid, and caffeic acid demonstrated the highest to lowest inhibitory efficacy against *Candida* species, correspondingly [152].

**Table 6:** This table summarizes the antimicrobial, antiviral, and antifungal activities of caffeic acid reported in experimental studies. The findings demonstrate that caffeic acid exhibits broad-spectrum inhibitory effects against bacteria, viruses, and fungi through multiple mechanisms including inhibition of biofilm formation and disruption of microbial membranes, inhibiting nucleic acid synthesis, and reducing biofilm formation.

Activity	Study Type	Findings	Refs.
Antibacterial	In vitro	° CA inhibited the MrsA pumps in the RN-4220 strain and the NorA strain 1199B	[143]
	In vitro	° CA alone proves antibacterial action and has the ability to enhance the antimicrobial effect when combined with antibiotics.	[145]
	In vitro	° CA was found to disrupt the biofilm formation of *E. coli* B1	[146]
	In vivo	° CA lessened the histological changes induced by *E. coli*.	[146]
Antiviral	In vitro	° Caffeic acid was virucidal against Ilhéus virus (ILHV)	[24]
	In vitro	° Caffeic acid effectively inhibits the multiplication of influenza A virus	[148]
	In vitro	° Caffeic acid inhibited HBV-DNA replication as well as HBsAg production.	[149]
	In vitro	° It reduced serum DHBV level in DHBV-infected duckling model	[149]
	In vitro	° Caffeic acid effectively inhibited CDV infection	[150]
	In vitro	° Caffeic acid inhibited the multiplication of HSV-1	[151]
Antifungal	In vitro	° Caffeic acid, nano-caffeic acid, carrier, fluconazole, and nystatin had the lowest to highest antifungal activity	[152]

## 5. Synergistic Effect of Caffeic Acid with Other Compounds

Natural compounds, including flavonoids and phenolic compounds, exhibit synergistic effects when combined with other phytochemicals or drugs by modulating various biological activities. Moreover, superior antimicrobial and anticancer activity was observed, including greater inhibition of biofilm formation, destruction of microbial growth, inhibition of cell proliferation, induction of apoptosis, and modulation of cell signaling pathways.

Enhanced biological activity, improved absorption, and increased bioavailability of caffeic acid have been noticed when combined with other compounds. For instance, when combined with compounds/drugs like curcumin, quercetin, and cisplatin, they may have improved potency (Table 7). The impact of combining CA with caffeine (CAF) was studied in relation to markers of toxicity in the male reproductive system. At a dosage of 50 mg/kg, CA and CAF confirmed a synergistic effect, leading to increased activity of testicular steroidogenic enzymes, as well as elevated levels of glycogen and zinc, while reducing testicular cholesterol. This combination therapy resulted in increased levels of follicle-stimulating hormone and luteinizing hormone. Additionally, it suppressed nitric oxide production in testicular cells, which further contributed to the downregulation of CAT and superoxide anions that are linked to lipid peroxidation [127].

A study was done to observe the ability of Lambda-cyhalothrin (LTC) to induce oxidative stress response in rat erythrocytes in vitro and the role of CA (20 μM) and/or quercetin (10 μM) in inhibiting cytotoxic activity. It was noticed that a combination of both compound pretreatments reduced the levels of lipid peroxidation markers and protein carbonyls (PCO), and DNA damage decreased in LTC portions [153]. Another research study focused on investigating the antioxidant-like properties of caffeic acid and rofecoxib in relation to QA-induced oxidative stress, mitochondrial impairment, and histological changes. Administration of caffeic acid (5 and 10 mg/kg) and rofecoxib (10 and 20 mg/kg) suggestively mitigated oxidative damage and enhanced mitochondrial enzyme activity in the ex vivo striatum. Additionally, the combination of sub-effective doses of rofecoxib (10 mg/kg) and caffeic acid (5 mg/kg) confirmed enhanced protective effects, resultant in a noteworthy improvement over their individual applications [154].

A research study inspected whether the combination of caffeic and ferulic acids offers greater benefits for individuals with metabolic syndrome. The findings showed that this treatment efficiently prevented weight gain and improved conditions such as hypertriglyceridemia, hyperglycemia, and hypercholesterolemia [155]. Another study based on cervical cancer reported that CA and metformin (Met) increased cisplatin effects against quiescent tumor cells involving reprogramming of the cell cycle [88]. CA and all-trans-retinoic acid (ATRA) combination was checked for the anti-neoplastic effect in Saos-2 as well as OSA-01, osteosarcoma cell lines. The outcomes exhibited the potential of CA in enhancing the anti-proliferative role of ATRA [156]. 

Another study reported that chlorogenic acid (ChA), apigenin (API), and caffeic acid (CaA) reduced CCl_4_-induced hepatotoxicity, particularly when administered in combination. Factorial analysis demonstrates that the three compounds showed the most potent synergistic effect in protecting against the CCl4-induced decrease in antioxidative activity [157]. Caffeic acid (CA) inhibited the growth of H1299 NSCLC cells by triggering apoptosis, and the combination of paclitaxel (PTX) and CA resulted in a synergistic anti-cancer effect in these cells [74]. Caffeic acid (CA) and cyclen-Zn(II) were combined into a G4 assembly using a phenylborate linker to form a mixed supramolecular prodrug hydrogel (GB-CA/Cy-Zn(II)). Analysis of typical inflammatory cytokines and in vitro antibacterial analyses confirmed that the hydrogel treatment efficiently suppressed both inflammation as well as infection. Additionally, in an in vivo infected wound model, the hydrogel exhibited excellent biosafety and meaningfully rapid wound healing [158]. The study examined the synergistic cytotoxic role of CA and benzyl isothiocyanate (BITC) in breast adenocarcinoma cells (MCF-7). It was reported that the combination selectively improved cancer cell death. Mechanistic investigations confirmed MAPK pathway activation as well as induction of apoptosis, supported by altered expression of GST, p-ERK, p38, Nrf-2, and ERK ½ and Bcl-2 proteins. Fluorometric examination demonstrates substantial disruption of redox homeostasis, together with changes in ROS, GSH, and caspase-3/7 activity. These outcomes highlight the synergistic effect of CA and BITC to modulate the MAPK pathway, disturb cellular homeostasis, and cause apoptosis induction, highlighting their ability for combination cancer therapy [159]. The study delivers more comprehensive information about the mechanisms of the improvement of the ATRA-induced differentiation of medulloblastoma cells. The expression profiling of the selected cancer-related genes evidently established that the differentiating effects of ATRA enhanced through its combined administration with CA or celecoxib. An increased genes expression of that encoded the proteins contributing in induced differentiation as well as cytoskeleton remodeling was noticed in both cell lines in a concentration-dependent way [160]. Combination therapy using CA and cisplatin has demonstrated promising anti-cancer effects in ovarian cancer. In cisplatin-sensitive cells treated with a combination of 5 µM cisplatin and 50 µM CA, there was a notable increase in apoptotic activity, indicated by a 1.7-fold rise in caspase activity compared to treatment with cisplatin alone. Additionally, research involving A2780cisR tumor cells revealed that the cisplatin/CA combination at a ratio of 5:50 µM led to a 4.3-fold increase in caspase activity, while maintaining 60% cell viability [161]. Another study aimed to investigate the effects of pioglitazone, caffeic acid, and their combination on chronic fatigue-like conditions in mice. In a three-week period, pretreatment with pioglitazone (at doses of 5 and 10 mg/kg) and caffeic acid (at doses of 5 and 10 mg/kg) reduced the symptoms of a chronic fatigue-like condition. Additionally, treatment with these drugs for three weeks notably decreased oxidative damage, altered the activities of mitochondrial enzyme complexes, and decreased mitochondrial redox activity. Moreover, the combination of the lower doses of pioglitazone (5 mg/kg) and caffeic acid (5 mg/kg) exhibited considerable synergy in their protective effects, which was greater than the effects observed when each was administered individually [162].

Another study’s results demonstrate that both 18 β-glycyrrhetinic acid and caffeic acid, whether administered separately or together, exhibit antidiabetic effects along with strong antioxidant properties in diabetic rats. The results indicate that combining these two compounds in diabetic rats produced a promising effect, enhancing antioxidant activity compared to using each treatment individually [58].

**Table 7:** Biological Activities of caffeic acid (CA) in combination with other compounds. The table presents the co-administered compound, type of biological activity investigated, and key experimental outcomes.

Compounds	Activity	Study Type	Outcomes	Refs.
Caffein	Improvement in male reproductive function	In vivo	° Combination treatment increased activity of testicular steroidogenic enzymes It reduced testicular cholesterol	[127]
Quercetin	Antioxidant	In vitro	° CA as well as quercetin pretreatments prevent the toxic effects of Lambda-cyhalothrin	[153]
Fosfomycin	Anti-microbial	In vitro	° The combined effect of CA with fosfomycin caused the inhibition of growth	[163]
Curcumin	Lung protective effect	In vivo	° Lung-protective activity was determined by combination of CA as well as curcumin	[164]
Ferulic acids	Metabolic syndrome prevention	In vivo	° The treatment with CA well as ferulic acids improved hyperglycemia, hypertriglyceridemia, and hypercholesterolemia	[155]
Cisplatin	Augment anticancer activity	In vitro	° Met as well as CA augmented cisplatin action against quiescent tumor cells	[88]
Apigenin, chlorogenic acid,	Hepaprotective effect	In vivo	° Chlorogenic acid, apigenin, and caffeic acid reduced hepatotoxicity	[157]
Paclitaxel	Anticancer effect	In vitro	° Co-administration of CA and PTX resulted in reduced cancer cell growth	[74]
Paclitaxel	Anticancer effect	In vivo	° Combination of CA and PTX caused better effective suppressive effect on tumor growth	[74]
Cisplatin	Anticancer effect	In vitro	° Cisplatin-caffeic acid inhibited cell growth of cervical cancer cells	[87]
Benzyl isothiocyanate	Anticancer effect	In vitro	° Study described synergistic potential of CA as well as BITC to modulate the MAPK pathway and apoptosis induction, highlighting their capacity for combination cancer therapy	[159]
All-trans retinoic acid	Anticancer effect	In vitro	° CA synergistic effect on ATRA in medulloblastoma cell lines was noticed	[160]

## 6. Role of Nano-Formulations of Caffeic Acid in Preclinical Studies and Translational Challenges

**Figure 7:**
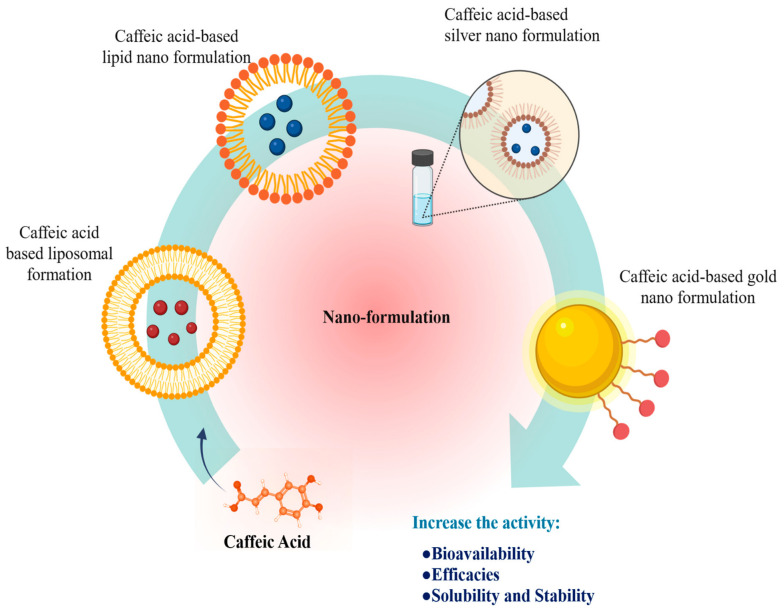
Nano-formulation strategies of caffeic acid to enhance therapeutic efficacy improved solubility and stability. These include caffeic acid–based liposomal formulations, lipid-based nanocarriers, and metal nanoparticle systems. These nanotechnology-based systems are designed to improve bioavailability, increase therapeutic efficacy, and improve solubility and stability. The figure was created using BioRender assessed on 13 January 2026. Rahmani AH. (2026). https://app.biorender.com.

Caffeic acid is an important compound in terms of health benefits through modulation of various biological activities. Despite its health benefits, the limitation of caffeic acid is its lower bioavailability due to rapid metabolism and conjugation in the body, poor solubility, and absorption, which may significantly decrease its systemic availability and biological efficiency. Nano-formulations of natural bioactive compounds, including flavonoids and phenolic compounds, based on nanoemulsions, micelles, liposomes, and gold/silver nanoparticles, are synthesized to overcome the limitations of poor solubility, low bioavailability, and rapid metabolism and enhance their therapeutic efficacy in different pathological conditions. Nano-formulations based on caffeic acid have been shown to enhance its solubility and stability (Figure 7).

Nano-formulations enhance the therapeutic effectiveness of drugs with poor oral bioavailability by improving solubility, increasing bioavailability, minimizing side effects, and extending circulation time [165]. Diverse types of nano-formulations based on caffeic acid have been developed and implication in pathological conditions documented. A study was made to produce liposomes loading CA. The findings demonstrate that CA-loaded Tf-functionalized liposomes can inhibit Aβ aggregation and fibril formation and promote the disaggregating of mature fibrils. Therefore, this brain-targeted drug delivery system may offer a promising strategy for Alzheimer’s disease [166]. Solid lipid nanoparticles combined with caffeic acid were developed. The effects of caffeic acid-loaded solid lipid nanoparticles on MCF-7 cells were determined by MTT test as well as Annexin V-PI analysis. Solid lipid nanoparticles may thus serve as an effective delivery system for caffeic acid, and the formulation of these nanoparticles enhanced the effects of caffeic acid [167].

A study examined the antitumor properties of conjugated caffeic as well as folic acid combined with silver nanoparticles against induced Ehrlich tumors. The results revealed that NSFC possessed strong oncolytic activity, delaying tumor growth in mice with Ehrlich solid carcinoma. This effect was linked to both the promotion of apoptosis in MCF-7 and Hep-G2 cells and the regulation of oxidative balance [168]. QuCaNPs, consisting of quercetin and CAPE co-loaded in PLGA nanoparticles, were developed and examined for their effects on HT-29 cells, demonstrating increased anticancer activity [169]. The study describes the one-pot synthesis and characterization of chitosan-capped silver nanoparticles functionalized with quercetin and caffeic acid (CA), termed Ch/Q-Ag NPs and Ch/CA-Ag NPs. Both formulations were assessed for antibacterial and anticancer activities and exhibited prominent anticancer effects. Among them, Ch/Q-Ag NPs exhibited higher efficiency, particularly against U-118 MG cells. Moreover, both nanoparticle types established dose-dependent antibacterial activity against Gram-positive and Gram-negative bacteria [170]. Caffeic acid–derived carbon nanodots were synthesized via a one-step hydrothermal process. These nanodots retain certain structural features and biological functions of caffeic acid, while exhibiting strong biocompatibility, good water solubility, and high stability. In vitro experiments showed that at low concentrations, these nanodots significantly suppressed the growth of poorly differentiated papillary thyroid carcinoma cells. Remarkably, at a concentration of 16 µg/mL, they achieved an inhibition rate of about 79% in human thyroid cancer cells. Additionally, in vivo experiments confirmed that these nanodots effectively reduce tumorigenicity in xenografts of papillary thyroid carcinoma at notably low doses [171]. Gold nanoparticles were synthesized from gold ions using CA as a natural reducing agent. Their formation was confirmed through multiple spectroscopic and microscopic characterization methods. The resulting nanoparticles showed effective catalytic performance in the reduction of 4-nitrophenol to 4-aminophenol when sodium borohydride was present [172].

Although nano-formulation-based delivery systems have demonstrated substantial potential in improving solubility, bioavailability, and targeted delivery, several challenges still hinder clinical translation. Safety concerns remain a major limitation, as long-term toxicity, biodistribution, and possible accumulation of nanoparticles in non-target tissues require. The toxicity linked with nanoparticles, recognized as nanotoxicity, arises from numerous physiological reactions initiated through their interactions with biological components [173].

Numerous studies have investigated potential toxic effects on major organs, including the lungs, liver, brain, and kidneys [174,175]. The toxicological profile of nanoparticles is strongly influenced by their physicochemical properties such as particle size, morphology, and surface functionalization [176]. Among these, particle size is a critical determinant of toxicity [177]. Several nanoparticle types, including silica (SiO_2_-NPs), carbon-based nanoparticles, silver (Ag-NPs), and zinc oxide nanoparticles (ZnO-NPs), have been reported to induce adverse effects, particularly in the respiratory system [178,179,180].

In addition to safety concerns, scalability represents significant barriers. Most caffeic acid-loaded nanoparticle systems remain at the laboratory scale and face challenges in large-scale manufacturing. Regulatory barriers further complicate translation, as nano-formulations require extensive characterization and comprehensive toxicological evaluation.

Moreover, the clinical feasibility of caffeic acid nano-formulations remains uncertain due to limited studies, insufficient pharmacokinetic data, and efficacy evidence. Moreover, the clinical feasibility also remains limited because evidence is predominantly based on vitro and preclinical studies. Although many studies have described enhanced therapeutic efficacy, but there is still inadequate evidence regarding long-term pharmacokinetics, biodistribution, therapeutic outcomes, and safety. From a formulation perspective, various nanocarrier systems have been developed to enhance the therapeutic performance of caffeic acid, each with distinct advantages and limitations. Among these, lipid-based nanocarriers and nanostructured lipid carriers appear promising, as they offer a favorable balance of biocompatibility, stability, and translational potential compared with metallic or inorganic nanoparticle systems.

## 7. Clinical Trials Based on Caffeic Acid

The role of caffeic acid has gained attention due to its multi-layered properties, with preclinical evidence from in vitro and in vivo studies indicating possible biological activity. Despite these promising results, the clinical trials examining its efficacy in human populations remain restricted due to poor bioavailability, pharmacokinetics, rapid degradation, toxicity, and differences in the tumor microenvironment. The lack of inclusive clinical data hinders the capability of this compound to draw definitive conclusions about the therapeutic role and mechanisms of action in pathological conditions. To completely understand its role in diseases, further research is indispensable to assess its safety profile and determine the optimal dosage. A study assessed the effectiveness and safety of oral caffeic acid (CA) tablets in management of primary immune thrombocytopenia (ITP). A total of 103 ITP patients with a PLT greater than 10 × 10^9^/L and without severe bleeding symptoms were recruited from three different centers. Based on their PLT prior to CA treatment, the patients were categorized into two groups: Group A, with PLT less than 30 × 10^9^/L, and Group B, with counts of 30 × 10^9^/L or higher. Participants in both groups were given CA tablets orally at a dosage of 300 mg thrice daily for 12 consecutive weeks. The outcomes indicated that treatment with CA was effective and linked with less side effects. Two cases of mild adverse effects were noticed: one patient experienced mild nausea, whereas another showed an elevation in liver enzymes. Both were grade 1 and transient [181]. Moreover, a meta-analysis was conducted of a total of 35 publications with an overall 2533 patients included. The results of the meta-analysis showed that caffeic acid tablets (CFA) were effective in thrombocytopenia treatment and in increasing platelet counts, neutrophil count, and white blood cell count, and CFA reduced myelosuppression. CFA has the ability to significantly enhance the clinical outcomes for patients suffering from thrombocytopenia with a good safety profile and is worth promoting [182].

## 8. Conclusions, Limitations, and Future Prospectives

Natural compounds and their bioactive components have been shown in preclinical studies to play a vital role in disease-related processes. Moreover, flavonoids and phenolic compounds are major classes of plant-derived phytoconstituents found in fruits, vegetables, and tea, known for their roles in health management. Among the phenolic compounds, caffeic acid, a hydroxycinnamic acid found widely in fruits, vegetables, coffee, and medicinal plants, has attracted substantial scientific interest due to its broad range of biological activities. It demonstrates its role through multiple molecular mechanisms, including scavenging reactive oxygen species, modulating inflammatory signaling pathways, inhibiting lipid peroxidation, and regulating cellular signaling proteins involved in apoptosis and immune responses. This review studies the role of caffeic acid in various health conditions through modulation of various biological activities. This review aims to summarize the evidence on the therapeutic role of caffeic acid across numerous pathologies, with particular emphasis on its antioxidant and anti-inflammatory mechanisms and their implications for disease management. By investigating the multifaceted roles of caffeic acid in these processes, we can better comprehend how this compound can contribute to health and possibly deliver new approaches for treating numerous pathological conditions. This review also discusses the anticancer effects of caffeic acid, including its mechanisms of action, such as inhibition of inflammation and angiogenesis, cell cycle arrest, and induction of apoptosis. Additionally, its synergistic role with other compounds is thoroughly examined. Caffeic acid has been shown in preclinical studies to play a significant role in disease-related processes; however, its clinical applicability is limited by poor bioavailability, pharmacokinetics, and low solubility. These issues lead to poor absorption and rapid metabolism. Toxicological research using animal models is required to improve understanding of safe dosing levels. Additionally, integrating nanotechnology into the formulation of caffeic acid is required to overcome the limitations of bioavailability and solubility, paving the way for its effective use in health management Additional comprehensive studies, together with in vivo investigations and clinical trials, are vital to assess its efficiency, illuminate its mechanisms of action, improve knowledge of its bioavailability, and explore its synergistic role with other compounds in in disease management.

## Figures and Tables

**Table 1:** Sources and content of caffeic acid (CA) in milligrams (mg) per 100 g.

Food Items/Sources	CA mg/100 g	References.
Potato cooked with peel	40	[28]
Carrot	26	[28]
Pot-grown basil	21	[28]
Jerusalem artichokes	21	[28]
Radish	1	[28]
Coffee	87	[29]
Black tea	1.48	[29]
Apple juice	~3.6	[29]
Orange juice	~2.5	[29]
Plum (dark)	~23.5	[29]
Cherry	17	[29]
Grapefruit	5.5	[29]
Buckwheat grits	8.5	[30]
Rye bran	7.7	[30]

**Table 3:** Neuroprotective effect of caffeic acid. Collectively, studies support the therapeutic role of caffeic acid in neurodegenerative diseases. It improved cognitive function, learning deficits, attenuated behavioral, biochemical and histopathological changes.

Study Model	Dose	Findings	Refs
Aβ-induced AD mice model	50 g/kg	° Spatial learning, cognitive abilities and memory improved upon CA administration ° CA enhanced synaptic markers	[63]
Dexamethasone induced depression in mice model	50 mg/kg	° CA showed antidepressant-like effect by reducing the hippocampal glutamate level	[64]
Neurogenesis and cognitive impairment in a rat model	20, 40 mg/kg	° Deficits in spatial memory prevented by CA	[65]
CdCl_2_-induced neurotoxicity rat model	10, 20 mg/kg	° CA administration improved cognitive function	[66]
L-methionine induced memory deficits in rats	20, 40 mg/kg	° CA protects memory deficits	[67]
Alzheimer’s disease rat model	100 mg/kg	° CA improved learning deficits and enhanced cognitive function ° It reduces acetylcholinesterase activity	[68]
Aluminium chloride-induced dementia rat model	100 mg/kg	° CA improved cognitive function	[69]
Streptozotocin-mediated dementia rat model	10, 20, 40 mg/kg	° CA treatment attenuated behavioral and biochemical abnormalities	[70]
Rotenone-induced neurodegeneration mice model	30 mg/kg	° Dopaminergic neurodegeneration prevented by CA	[71]
Pilocarpine-induced seizures rat model	4 mg/kg	° CA treatment decreases in lipid peroxidation and nitrite content	[72]

## Data Availability

No new data were created or analyzed in this study. Data sharing is not applicable to this article.

## Abbreviations

AChE	Acetylcholinesterase
AD	Alzheimer’s disease
BCHE	Butyrylcholinesterase
CAPE	Caffeic acid phenethyl ester
CA	Caffeic acid
CAT	Catalase
CDH2	Cadherin-2
CHA	Chlorogenic acid
COX-2	Cyclooxygenase-2
CDDP	Cisplatin
CYT C	Cytochrome c
DSS	Dextran sulfate sodium
DEXA	Dexamethasone
DIO	Diet-induced obesity
IM	Imatinib
INOS	Inducible nitric oxide synthase
MDA	Malondialdehyde
MRT	Mean residence time
MTF	Metformin
NO	Nitric oxide
PC	Prostate cancer
PCOS	Polycystic ovary syndrome
PGE**_2_**	Prostaglandin E_2_
PTX	Paclitaxel
ROS	Reactive oxygen species
STZ	Streptozotocin
VEGF	Vascular endothelial growth factor
